# Exhausted-like CD8^+^ T cell phenotypes linked to C-peptide preservation in alefacept-treated T1D subjects

**DOI:** 10.1172/jci.insight.142680

**Published:** 2021-02-08

**Authors:** Kirsten E. Diggins, Elisavet Serti, Virginia Muir, Mario Rosasco, TingTing Lu, Elisa Balmas, Gerald Nepom, S. Alice Long, Peter S. Linsley

**Affiliations:** 1 Systems Immunology, Benaroya Research Institute at Virginia Mason, Seattle, Washington, USA.; 2 Immune Tolerance Network (ITN), Bethesda, Maryland, USA.; 3 Translational Immunology, Benaroya Research Institute at Virginia Mason, Seattle, Washington, USA.

**Keywords:** Autoimmunity, Immunology, Adaptive immunity, Autoimmune diseases, T cells

## Abstract

Clinical trials of biologic therapies in type 1 diabetes (T1D) aim to mitigate autoimmune destruction of pancreatic β cells through immune perturbation and serve as resources to elucidate immunological mechanisms in health and disease. In the T1DAL trial of alefacept (LFA3-Ig) in recent-onset T1D, endogenous insulin production was preserved in 30% of subjects for 2 years after therapy. Given our previous findings linking exhausted-like CD8^+^ T cells to beneficial response in T1D trials, we applied unbiased analyses to sorted CD8^+^ T cells to evaluate their potential role in T1DAL. Using RNA sequencing, we found that greater insulin C-peptide preservation was associated with a module of activation- and exhaustion-associated genes. This signature was dissected into 2 CD8 memory phenotypes through correlation with cytometry data. These cells were hypoproliferative, shared expanded rearranged TCR junctions, and expressed exhaustion-associated markers including TIGIT and KLRG1. The 2 phenotypes could be distinguished by reciprocal expression of CD8^+^ T and NK cell markers (GZMB, CD57, and inhibitory killer cell immunoglobulin-like receptor [iKIR] genes), versus T cell activation and differentiation markers (PD-1 and CD28). These findings support previous evidence linking exhausted-like CD8^+^ T cells to successful immune interventions for T1D, while suggesting that multiple inhibitory mechanisms can promote this beneficial cell state.

## Introduction

Type 1 diabetes (T1D) is an autoimmune disease in which β cells of the pancreas are destroyed, resulting in a life-long dependence on exogenous insulin (1). As some residual β cell function is present upon diagnosis, the preservation of remaining β cell function is the primary therapeutic goal when treating recent-onset (RO) T1D. The goal of immunotherapies is to establish and maintain a tolerogenic immune state. While, to our knowledge, no therapies to date have preserved β cell function beyond 1 year in all treated RO T1D subjects (2–19), a subset of subjects respond better than others in many of these trials. Understanding immune states and immunological changes associated with treatment and outcome can help improve tolerance-inducing strategies that prolong clinical benefit.

An emerging mechanism in slower autoimmune disease progression (20) and better response to therapy is T cell exhaustion (21, 22). An expansion of TIGIT^+^KLRG1^+^ (double positive; DP) CD8^+^ T cells, described as partially exhausted, was linked to good clinical outcome in trials of anti-CD3 mAb teplizumab in RO and at-risk subjects in T1D (22). Other successful biologic therapies for T1D did not trigger obvious accumulation of DP cells (23, 24), nor were they observed in bulk transcript profiles from untreated subjects (25), demonstrating context specificity. However, exhausted autoreactive CD8^+^ T cells were also linked to rate of progression in established T1D (26).

Alefacept is a LFA-3-Ig fusion protein that binds CD2 (27), disrupts CD58-mediated costimulation of T cells (28), and selectively depletes memory/effector T cells (29–33) via NK-mediated antibody–mediated cytotoxicity (ADCC) (31). The T1DAL study was a phase 2, double-blind, placebo-controlled trial of alefacept in RO T1D patients diagnosed within 100 days leading up to enrollment in the trial. Alefacept resulted in significant preservation of endogenous insulin production in 30% of treated subjects after 2 years compared with placebo (11, 15). Alefacept treatment altered immunological profiles and induced potentially tolerogenic changes in the T cell compartment. CD4 effector memory and central memory T cells (TEM and TCM cells, respectively) were depleted and CD4^+^FOXP3^+^CD127^lo^ Tregs were preserved, resulting in an increased ratio of Tregs to memory T cells.

Given that exhausted CD8^+^ T cells (TEX cells) have been associated with response to other T cell targeting therapies (22), we explored the possibility that alefacept had immunomodulatory effects on residual CD8^+^ T cells by performing a detailed analysis of the phenotype and function of CD8^+^ T cells following therapy and relating their biological signatures with clinical outcome. Using integrated RNA sequencing (RNA-seq) and CyTOF analysis, we identified and describe 2 CD8 phenotypes that correlated with clinical response: one, whose frequency was maintained and whose phenotype was defined by higher CD57 expression, and another, whose frequency recovered after therapy in treatment responders and whose phenotype was defined by higher PD-1 expression. These cells were phenotypically distinct from one another, but both shared characteristics of the DP cells previously seen in responders to teplizumab (anti-CD3) in the AbATE trial (22), including high inhibitory receptor (IR) expression and hypoproliferation following anti-CD3/anti-CD28 stimulation. These results expand on the evidence that therapeutic modulation of CD8 populations with features of exhaustion or terminal differentiation are linked to preservation of β cell function in T1D.

## Results

Alefacept was shown to deplete CD2^hi^ CD4^+^ and CD8^+^ TEM and TCM subsets in all subjects in the T1DAL trial; however, these changes were not associated with therapy response as measured by maintenance of β cell function (15). While most T cell populations were depleted consistently across all subjects, the CD8^+^ TEM cells showed notable variability in the extent of depletion, suggesting that differential changes related to response could exist in this compartment. Additionally, previous evidence indicated that expansion of CD8^+^ TEX cells was linked to better outcomes in T1D clinical trials of biologic agents (22). We therefore hypothesized that phenotypic and functional changes occurring after treatment in T1DAL subjects’ CD8^+^ T cells would identify biologically and therapeutically relevant signatures of response.

**Figure 1:**
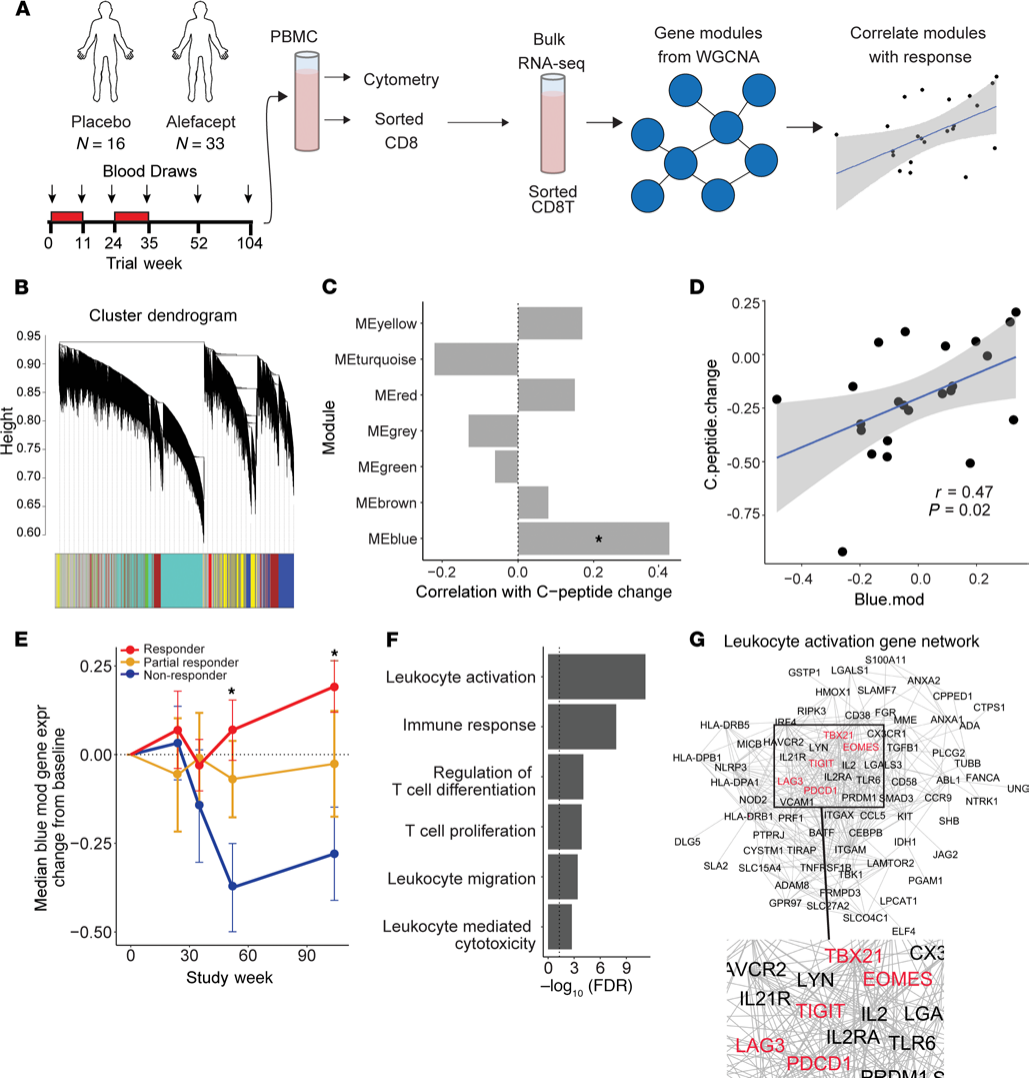
CD8^+^ T cell activation and exhaustion-associated gene signature was associated with response to alefacept. (**A**) Schematic diagram shows analysis workflow. (**B**) WGCNA cluster dendrogram is shown for analysis of 5000 most variable genes in CD8 samples (*n =* 24; including 2 placebo). (**C**) Pearson correlation between module eigengene and C-peptide change. Significance of correlation was determined by Student’s asymptotic 2-tailed *t* test. Only the blue module was significantly correlated with C-peptide change (*P < 0.05). Correlation and significance calculations included all 24 subjects used for WGCNA module generation. (**D**) Graph shows blue module eigengene expression versus C-peptide change at week 104 across the same 24 subjects (*r* = 0.47, *P* = 0.023, FDR = 0.14). Pearson correlation and the corresponding 1-tailed *t* test of correlation significance were performed using cor.test function in R. (**E**) Change from baseline median expression of blue module genes in responders, partial responders, and nonresponders over time. See Supplemental Table 1 for sample numbers per group and visit. Significant differences at week 52 and 104 were seen between responders and nonresponders (*P < 0.05). Significance was determined by repeated-measures 1-way ANOVA, with multiplicity adjustment applied to *P* values. (**F**) A selection of significantly enriched terms identified by GO enrichment analysis of blue module genes are shown with their respective enrichment *P* value (–log_10_[FDR]). (**G**) Blue module genes categorized as “leukocyte activation” by GO analysis were clustered using string (string-db.org) and visualized in Cytoscape. Inhibitory marker names colored red.

To address this hypothesis, we obtained frozen peripheral blood mononuclear cells (PBMCs) that were isolated from trial subjects at 5 time points: immediately before treatment, twice during the course of treatment, and twice up to 2 years after treatment (Figure 1A). As previously described (11, 15), 49 RO T1D subjects were enrolled and randomly assigned to receive alefacept (*n =* 33) or placebo (*n =* 16). Sorted CD8^+^ T cells from 30 of these subjects were analyzed by bulk RNA-seq. Following quality control and filtering, transcript data from 26 of these subjects were included in gene expression analysis. Additionally, PBMCs from treated and placebo subjects were analyzed by flow and mass cytometry (Table 1). Subjects were selected to maximize response variability and, therefore, included subjects with the greatest C-peptide preservation (responders) or loss (nonresponders) at week 104. Neither the RNA-seq nor the cytometry cohorts differed significantly from the original cohort of 33 subjects in terms of age, sex, and response (Table 1 and ref. 15).

### A CD8^+^ T cell activation and exhaustion-related gene signature was associated with response to alefacept.

We applied weighted gene coexpression network analysis (WGCNA) to postprocessed gene counts in order to discover modules of coregulated genes via an unsupervised approach (34). WGCNA is an unbiased clustering method that identifies sets, or modules, of correlated genes with the assumption that genes whose expression is highly correlated are likely involved in the same biological functions or pathways. We reasoned that WGCNA would identify immunological pathways or functions that were linked to therapy response. The analysis included samples from the end of the trial (2 years after treatment) given that the greatest disparity existed in outcomes at this time point. This also enabled evaluation of long-term remodeling of the CD8^+^ T cell compartment that might point to persisting, long-term alterations related to better response.

We performed WGCNA on the top 5000 most variable genes across all available week 104 samples (*n =* 24; Supplemental Table 1; supplemental material available online with this article; https://doi.org/10.1172/jci.insight.142680DS1). The analysis identified 6 distinct gene modules, ranging in size from 62 to 738 genes (Figure 1B). An additional module, labeled as the gray module by WGCNA, included the remaining, uncorrelated genes. Module eigengenes (weighted combination of module gene expression levels) were correlated with C-peptide change (defined as the slope from random-effects model fitted to 4-hour AUC from baseline through the end of the trial). Of these 7 modules, only the blue module was significantly correlated with response (*n =* 738 genes; correlation *P* < 0.05), indicating that higher expression of the genes in this module occurred in subjects with prolonged C-peptide preservation (Figure 1, C and D). Given that higher blue module gene expression was seen in those with better outcomes at the end of the trial, we wanted to evaluate if this difference occurred only 2 years after treatment or showed divergence between groups earlier in the trial. To evaluate the dynamics of blue module expression over the course of the trial, change from baseline median module gene expression was determined for all available samples per visit (Supplemental Table 1 and Figure 1E). Median blue module gene expression declined in nonresponders at week 52, while remaining higher in responders over the course of the trial (Figure 1E). This module was not correlated with age (Supplemental Figure 1), indicating that increased expression of these genes was not the result of an age-related immune state.

Gene ontology (GO) analysis was applied to the blue module to identify enriched functional pathways within the gene set. Of the 837 significantly enriched GO terms (FDR < 0.01), 114 terms were related to T cell activation, differentiation, cytotoxicity, and apoptosis (Figure 1G). These terms included the genes *CD38*, *EOMES*, *GZMB*, *TIGIT*, *LAG3*, *KLRD1*, and *CD160*, among other IR genes and genes associated with T cell activation responses (Figure 1G). Because of the high diversity of functional pathways associated with blue module genes, we hypothesized that the response signature could be arising from more than one CD8^+^ T cell population.

### Increased frequency of CD8^+^ TEM cell populations with high IR expression was associated with beneficial clinical response to alefacept.

**Figure 2:**
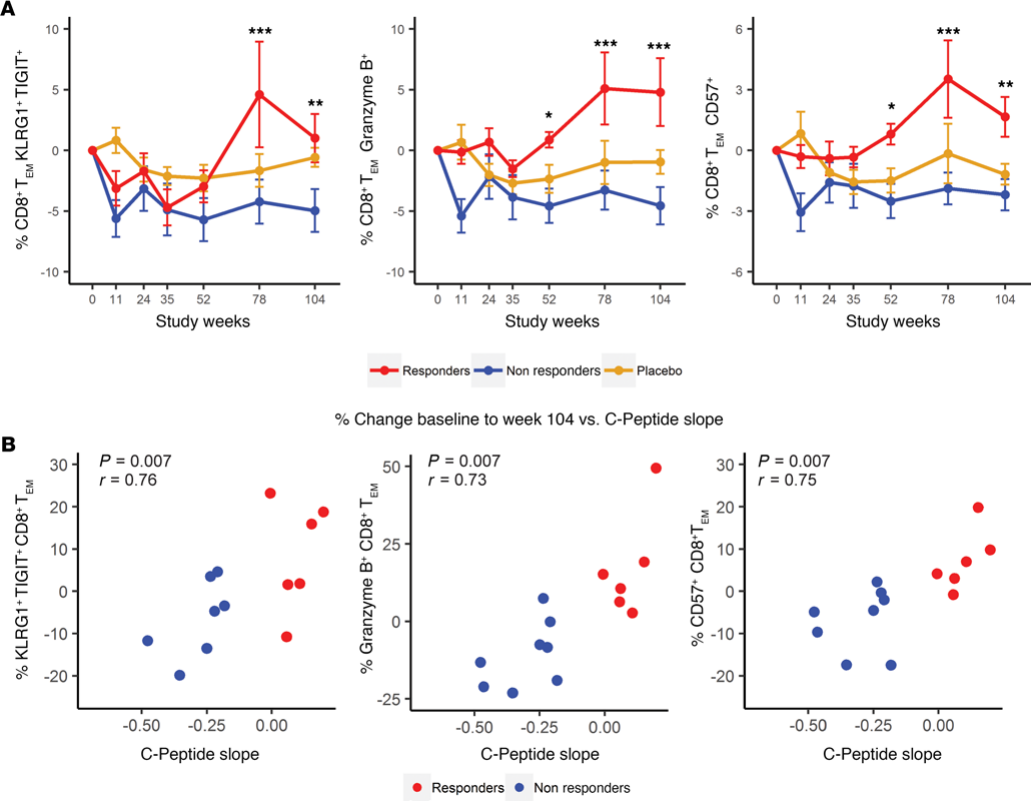
Increased frequency of memory CD8 subsets correlated with beneficial response to alefacept. (**A**) Longitudinal analysis of KLRG1^+^ΤIGIT^+^, Granzyme B^+^, and CD57^+^ is shown as the change from baseline percentage of CD8^+^ TEM in responders (*n =* 7; red), nonresponders (*n =* 9; blue), and placebo (*n =* 12; yellow). Differences between groups were analyzed by repeated-measures 1-way ANOVA with baseline adjustment and Bonferroni multiple comparison correction.*P < 0.05, **P < 0.01, ***P < 0.001. C1, cycle 1 of treatment; C2, cycle 2 of treatment. (**B**) Plots show correlation of the C-peptide change with the change of %KLRG1^+^TIGIT^+^, Granzyme B^+^, and CD57^+^ CD8^+^ TEM from baseline to week 104 in responders (*n =* 6) and nonresponders (*n =* 7). Spearman correlation were performed with FDR adjustment for multiple comparison.

**Figure 3:**
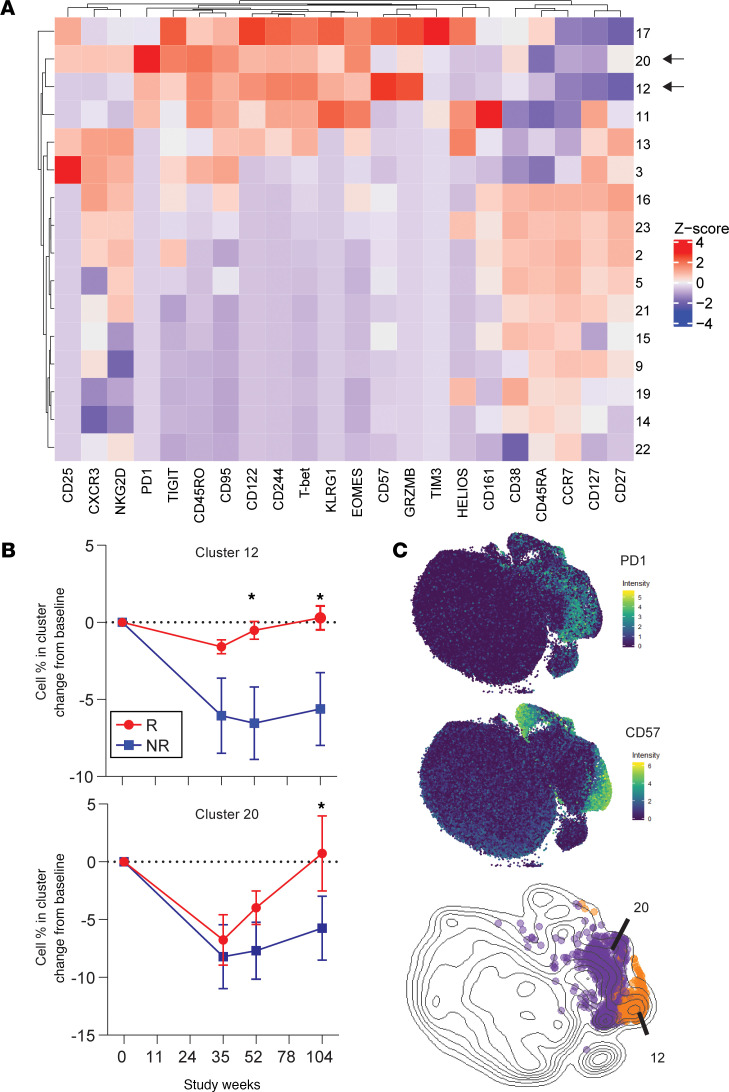
Two CD8^+^ memory T cell subsets identified by CyTOF analysis were associated with beneficial response to alefacept. (**A**) Heatmap shows median expression for each cluster that contained at least 1% of total cells. Color indicates column *Z*-score calculated from cluster median expression. Arrows denote the 2 IR-high clusters that correlated with response, clusters 12 and 20. Analysis included 12 subjects (6 responders [R]; 6 nonresponders [NR]). (**B**) Change from baseline percent of cells in clusters 12 and 20 plotted over time in 6 R and 6 NR. Differences between groups were analyzed by repeated-measures 1-way ANOVA with baseline adjustment and FDR multiple comparison correction. *P < 0.05. (**C**) t-SNE was used to visualize CyTOF data at a single-cell level. Expression intensity (asinh of MI) is colored on t-SNE plots for PD-1 and CD57 (top). Overlay of cluster 12 and 20 cells shown on density plot, color-coded as orange and purple, respectively (bottom).

First, we evaluated the CD8^+^ TEM cell populations expressing cytotoxic and exhaustion-associated markers by flow cytometry analysis (Supplemental Figure 2), given that the gene signature identified by WGCNA included many IR genes, as well as previous evidence that exhausted-like cells have been associated with immunotherapeutic response in T1D (22). Within the CD8^+^ TEM cell compartment (CD45RO^+^CCR7^–^; Supplemental Figure 2), there was an increased frequency of cells that were KLRG1^+^TIGIT^+^, CD57^+^, or Granzyme B^+^, with the highest frequencies found in subjects with favorable clinical outcomes (Figure 2A). The change in frequency of these cells from baseline to the end of the trial correlated with the rate of change of C-peptide in treated subjects (Figure 2B) but not in placebo subjects (Supplemental Figure 3), suggesting that maintenance or relative increases in these CD8^+^ TEM cell phenotypes may contribute to the role of alefacept in preserving β cell function. This association with response was specific to CD8^+^ T cells, as neither the change in total NK cell frequency (CD3^–^CD56^+^) nor CD57^+^ NK cell frequency (CD3^–^CD56^dim^CD57^+^) differed between response groups (Supplemental Figure 4). In other experiments, we investigated whether PD-1^+^CD8^+^ TEM cell frequencies would also stratify subjects by treatment response and found that these cells did not significantly differ over the course of the trials between placebo, responder, and nonresponder groups. To further refine and characterize the CD8 subsets associated with response, we performed high-dimensional CyTOF analysis of activation, differentiation, and exhaustion markers on total CD8^+^ T cells in 12 alefacept-treated subjects, followed by unsupervised clustering and data visualization with Rphenograph and t-SNE. Of the 23 clusters defined by Rphenograph, 16 of them contained at least 1% of the total cells and were included in subsequent analyses. Hierarchical clustering of the subsets according to marker intensity revealed the presence of multiple naive and memory-like CD8 clusters, including populations expressing high levels of IRs like PD-1, likely corresponding to subsets of the response-associated, IR-high CD8^+^ TEM cells seen by flow cytometry (Figure 2A and Figure 3A).

We compared frequencies of the 16 clusters between response groups over time through the end of the trial (Supplemental Figure 5). An initial decrease was seen in the frequencies of several memory CD8 clusters across both response groups. For example, cluster 3 (CD25^+^CD127^+^), cluster 1 (KLRG1^+^CD161^+^), and cluster 20 (PD-1^+^) decreased from baseline to week 35 across subjects. Several naive CD8 clusters, including cluster 9 (CD127^+^CCR7^+^) and cluster 15 (CD38^+^CCR7^+^), increased in frequency during the same time frame (Figure 3 and Supplemental Figure 5). These changes corresponded to the previously described depletion of TEM cells by alefacept and reflected an expected change in population proportions that occurred across all treated subjects in the weeks following therapy (11, 15).

While these broad, posttreatment alterations to the CD8^+^ T cell compartment occurred across subjects regardless of outcome, differences in the frequency changes of 2 IR-high clusters, clusters 12 and 20, were observed when comparing responders with nonresponders (Figure 2B and Supplemental Figure 5). Although nonresponders had significantly more cells in cluster 12 at baseline, the frequency of these cells declined following treatment in nonresponders but was maintained at approximately baseline levels in responders over time. Frequency of cells in cluster 20 declined in all subjects following therapy but recovered more extensively by week 104 in responders than nonresponders (Figure 3B). Baseline frequency of cells in cluster 3 (CD45RA^+^CD25^+^) was also associated with response (Supplemental Figure 5); however, this population declined in both treatment groups and did not significantly differ between them after treatment. We therefore chose to focus further analyses on clusters 12 and 20 due to their positive correlation with good response, which could indicate a mechanistic role for them in alefacept’s efficacy against T1D onset.

Both clusters 12 and 20 expressed CD45RO but not CD45RA, confirming their memory lineage, and they also expressed TIGIT, T-BET, KLRG1, CD244, CD122, and EOMES (Figure 3, A and C). Although both clusters grouped closely on the t-SNE map, forming a large cluster of TEM cells, they could be distinguished from one another by higher expression of CD57 and Granzyme B on cluster 12 and higher expression of PD-1 on cluster 20 (Figure 3A). Cluster 20 expressed moderate levels of CD27, which could indicate a transitional memory phenotype.

Cluster 17 expressed high levels of CD57 and GZMB, as well as moderate levels of exhaustion-associated markers TIGIT and CD244 (Figure 3A and Supplemental Figure 6). However, this cluster also expressed CD45RA and HELIOS, suggesting a more activated, TEMRA-like phenotype inconsistent with functional exhaustion. Cluster 11, another CD45RA^–^CD45RO^+^ memory CD8 cluster, expressed low levels of KLRG1 and CD244. However, expression of functional activation markers like HELIOS and CD161 suggests that cluster 11 was not functionally exhausted in spite of moderate expression of IRs and exhaustion-associated transcription factors. Clustering analysis, therefore, enabled us to distinguish clusters 12 and 20 from other IR-expressing and cytotoxic cells. Cluster 12 appeared to be a subpopulation of the CD57^+^ and GZMB^+^ TEM cells characterized by flow cytometry analysis, while Cluster 20 likely corresponded to a PD-1^hi^ subset of the TIGIT^+^KLRG1^+^ TEM cell population (Figure 2A).

Finally, we correlated the frequencies of clusters 12 and 20 with expression of blue module genes to determine if patterns of gene expression and cell frequencies supported the hypothesis that both the RNA-seq and cytometry response signatures were arising from the same populations of cells (Supplemental Figure 7 and Supplemental Figure 8). Positive correlations existed between the blue module gene expression and frequencies of clusters 12 and 20, as well as clusters 3 and 17 (Supplemental Figure 8). Significant correlations existed between other cluster frequencies and modules (Supplemental Figure 9); however, the lack of response associations between these modules and clusters led us to focus further analysis on the blue module and clusters 12 and 20.

Although only the correlation between cluster 20 and blue module gene expression reached significance, the trend toward a positive correlation supported the hypothesis that an elevated proportion of these cells likely contributed to the observed elevation in blue module gene expression in responders. However, these positive correlations were not exclusive to clusters 12 and 20. The diverse gene pathways represented in the blue module (Figure 1F) suggested that multiple cell types likely contributed to the total signature, which is supported by the positive correlations between blue module gene expression and frequencies of clusters 3 and 17, in addition to clusters 12 and 20 (Supplemental Figure 8 and Supplemental Figure 9).

Together, these results suggest that maintenance of CD8 cells with terminal, exhausted-like phenotypic signatures was linked to better therapy response.

### CD57^+^ and PD-1^+^ response-associated CD8^+^ T cells were hypoproliferative and expressed features of NK function and T cell activation.

**Figure 4:**
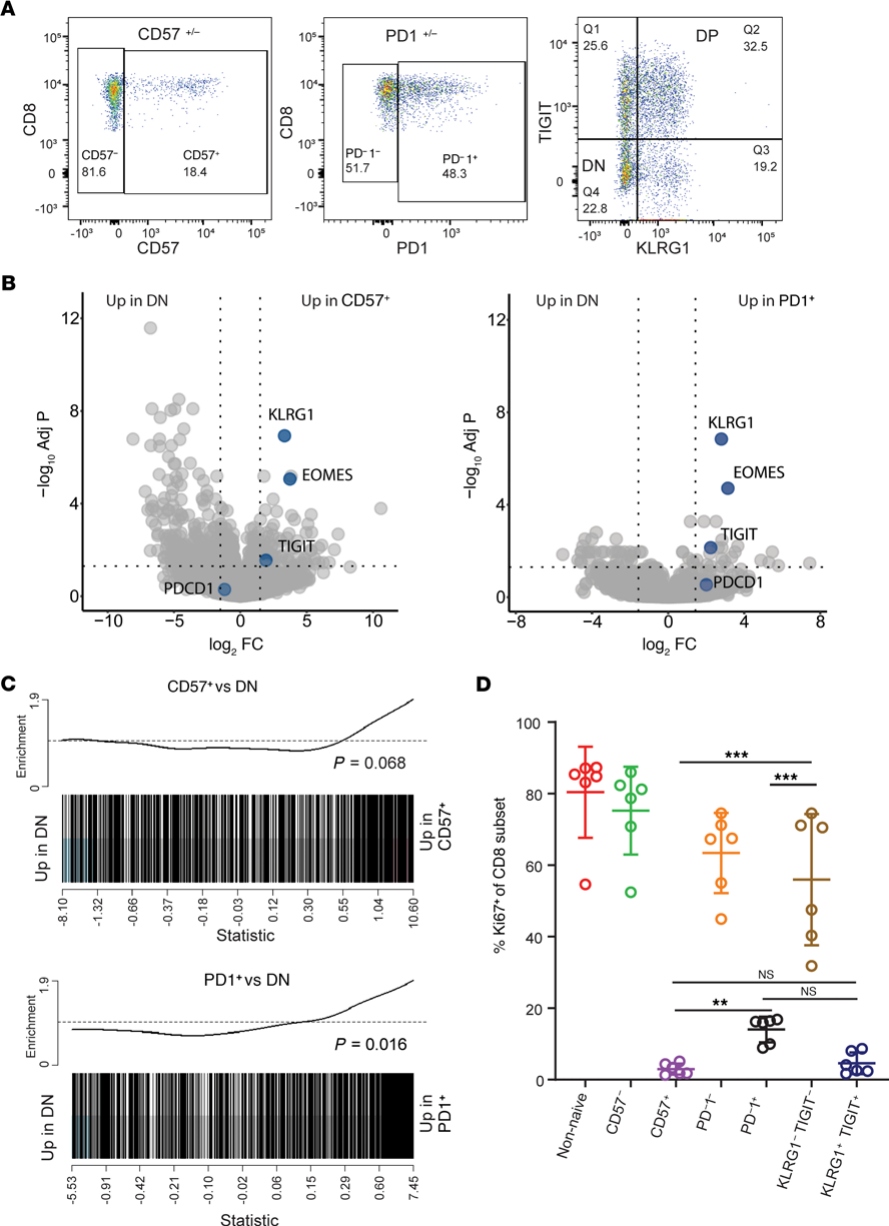
CD57^+^ and PD-1^+^ response-associated CD8^+^ T cells were hypoproliferative and expressed features of exhaustion. (**A**) Representative plots illustrate sorting strategy to isolate memory (nonnaive CD8 excluding CD45RA^+^CCR7^+^) CD8^+^ T cell populations (CD57^+/–^, PD-1^+/–^, and KLRG1/TIGIT^+/–^). (**B**) Volcano plots show differentially expressed genes contrasting PD-1^+^ (right) and CD57^+^ (left) with DN cells. Genes canonically associated with exhaustion, as well as key markers of clusters 12 and 20, are highlighted and labeled. (**C**) Enrichment barcode plots contrast CD57^+^ and PD-1^+^ populations with KLRG1/TIGIT–double negative cells in their expression of blue module genes. *P* value from rotation gene set analysis *roast* in R. (**D**) Ki67^+^ cell frequency in each of the sorted CD8 subsets (week 104 samples; *n =* 6) following 4 days of stimulation with plate-bound anti-CD3/soluble anti-CD28. Data are presented as mean ± SEM. *P* values were calculated using 1-way ANOVA, and results are displayed for comparisons of CD57^+^, PD-1^+^, and KLRG1^–^TIGIT^–^ (DN) frequencies. *P < 0.01, ***P < 0.001.

To further characterize the phenotype and function of cells in clusters 12 and 20, we defined these populations as CD45RA^–^PD-1^+^CD8^+^ (cluster 20) and CD45RA^–^CD57^+^CD8^+^ (cluster 12) (Figure 4A). Hypergate was used to confirm that these markers were the most likely to yield sorted subsets with the highest possible purity (Supplemental Figure 10; ref. 35). Sorting only CD45RA^–^CD8^+^ T cells excluded the naive CD57^+^CD45^+^ population (cluster 17) from the sorted CD57^+^ population. PD-1 was selected as the primary identity marker for cluster 20, given that its expression was a distinct feature of cluster 20, with only low expression on a small percentage of cells in clusters 11 and 12 (Figure 3, A and C, and Supplemental Figure 6). The upstream CD8 sort was performed as shown in Supplemental Figure 2.

To enable comparison with exhausted-like and nonexhausted cells, we also sorted 2 populations to serve as positive and negative controls for the exhaustion phenotype, as previously described (22). These included CD8^+^ memory T cells that coexpressed the IRs TIGIT and KLRG1 (DP) and CD8^+^ memory T cells that expressed neither of these receptors (double negative; DN). We then performed bulk RNA-seq, followed by differential gene expression analysis (DGEA) and rotation gene set testing on the sorted populations.

DGEA revealed higher expression of IRs and exhaustion-associated genes, including *KLRG1*, *TIGIT*, and *EOMES*, in both PD-1^+^ and CD57^+^ T cells relative to DN cells (Figure 4B). Also relative to DN cells, both PD-1^+^ and CD57^+^ T cells were enriched for blue module genes, confirming that these cells likely contributed to the gene expression response signature seen in the bulk CD8 RNA-seq analysis (Figure 4C).

To determine the extent to which these populations’ gene expression profiles aligned with profiles of DP cells, we defined the DP-associated gene set by contrasting our sorted DP and DN cells using limma and selecting genes whose expression was significantly higher in DP relative to DN cells (adjusted *P <* 0.05). We then used this set of DP-associated genes in an enrichment analysis comparing PD-1^+^ and CD57^+^ T cells with the DN population. Both populations were highly enriched for DP-associated genes relative to DN (Supplemental Figure 11). These results confirmed that both PD-1^+^ and CD57^+^ T cells have features associated with exhaustion, consistent with the high IR expression seen on clusters 12 and 20 by CyTOF analysis and elevated DP and CD57^+^CD8^+^ TEM cells seen by flow cytometry.

To directly test proliferative activity of PD-1^+^ and CD57^+^ T cells, we stimulated CD8 memory T cell subsets in vitro with anti-CD3 and anti-CD28 mAbs and measured Ki67 expression at day 4 as a measure of proliferation (Supplemental Figure 12 and Figure 4D). Both PD-1^+^ and CD57^+^ subsets were hypoproliferative relative to total memory, PD-1^–^, CD57^–^, and DN control populations (Figure 4D).

Because CD57 has canonically been used as a marker of senescence, we tested for enrichment of genes related to telomere maintenance, DNA replication, and senescence in the CD57^+^ relative to PD-1^+^ T cells (Supplemental Figure 13). We defined a telomere-associated gene set (*n =* 175) derived from overlap between the Molecular Signatures Database(MSigDB; http://www.gsea-msigdb.org/gsea/msigdb/index.jsp) “telomere organization” set and any REACTOME pathways. We also divided these genes into functionally distinct subsets including histones, polymerases, DNA repair, and replication for gene set testing. Enrichment analysis with *roast* showed that, of the total telomere-related gene set and the functional gene sets, only the polymerase genes were significantly enriched in either population, with increased enrichment in PD-1^+^ T cells relative to CD57^+^ T cells (*P* = 0.01). Two additional, previously defined gene sets associated with p53 and senescence were also tested for enrichment (36). Both gene sets showed low levels of enrichment in PD-1^+^ versus CD57^+^ T cells, with 1 set reaching statistical significance (Supplemental Figure 14). Together, these results indicate that, while the CD57^+^ T cells were hypoproliferative, they did not upregulate genes related to the processes involved in classical senescence.

### CD57^+^ and PD-1^+^ TEM cells shared a subset of TCRs but differentially expressed features of NK function and T cell activation.

**Figure 5:**
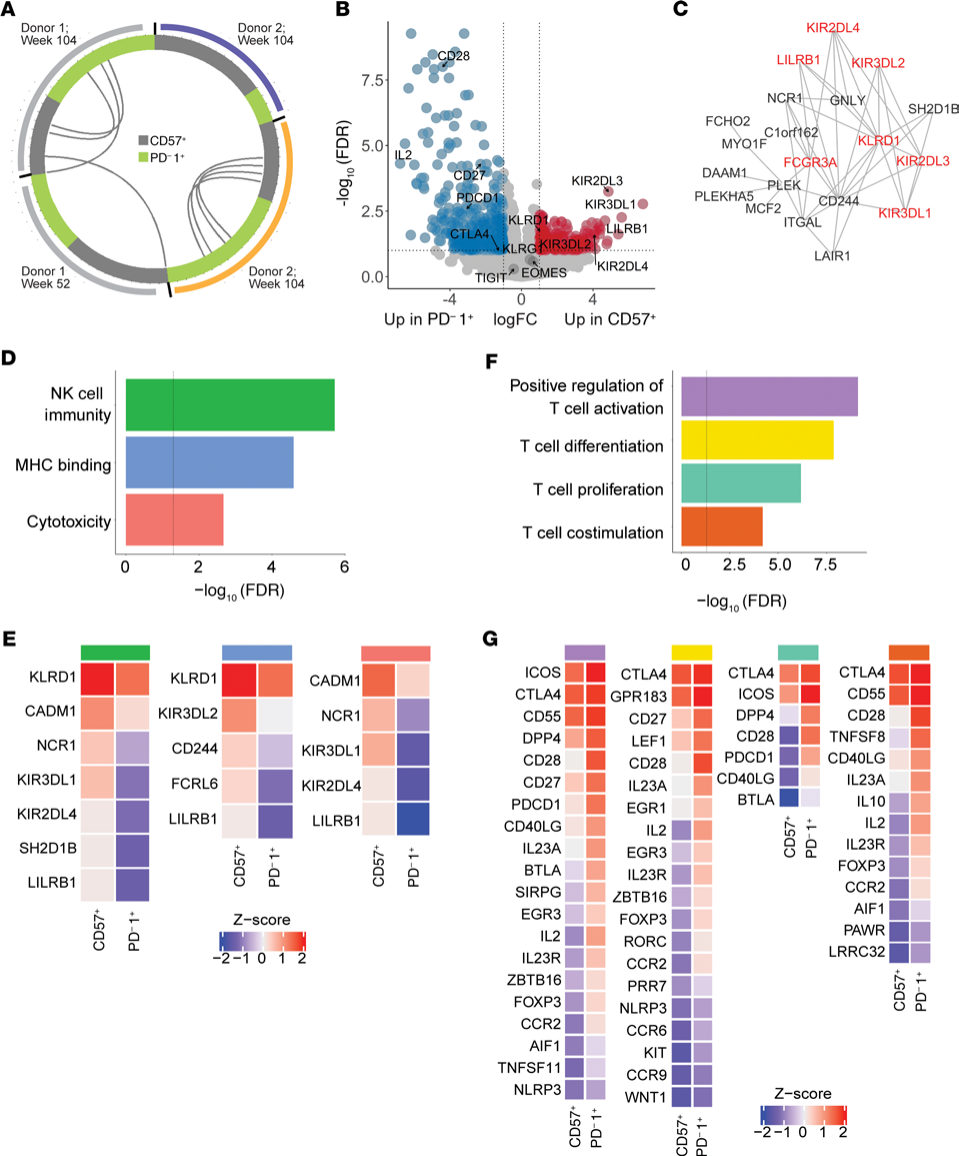
CD57^+^ and PD-1^+^ T cells shared a subset of TCRs but differentially expressed features of NK cytotoxicity and T cell activation. (**A**) Each segment of the Circos plot represents a TCR junction found in PD-1^+^ (green) or CD57^+^ (gray) cells from the 4 sorted samples. Color bars denote donors. Arcs connect junctions shared between samples. (**B**) Differentially expressed genes identified by limma analysis contrasting PD-1^+^ (left) and CD57^+^ (right) cells. Genes whose differential expression reached significance are highlighted in blue and red (log_FC_ > 1; FDR-adjusted *P <* 0.1). (**C**) Network shows connected network of genes expressed significantly higher in CD57^+^ than PD-1^+^ cells. Key NKRs are highlighted in red. (**D**) Selection of enriched pathways in CD57^+^ cells are shown with respective significance of GO term gene overrepresentation in set. (**E**) Relative expression of pathway-associated genes in CD57^+^ and PD-1^+^ cells, calculated as the mean scaled expression of the gene across all samples. (**F**) Selection of enriched pathways in the genes differentially expression in PD-1^+^ cells, determined as in **D**. (**G**) Relative expression of pathway-associated genes in PD-1^+^ and CD57^+^ cells, analyzed as in **E**.

To determine if these cells represented 2 clonally related states of exhaustion, we evaluated TCR sharing to identify potential clonal relationships. We adapted procedures we developed previously for single cell analysis for bulk RNA-seq (37, 38). In preliminary experiments, these procedures were able to accurately detect known, high-abundance rearranged TCR-α (TRAV) and -β (TRBV) chain complementarity-determining region 3 (CDR3) regions (junctions) in bulk T cell profiles. We applied these procedures to our bulk RNA-seq profiles from the sorted PD-1^+^ and CD57^+^ T cells. Circos plots linking rearranged junctions showed TCR junction sharing between CD57^+^ and PD-1^+^ T cells from 2 of 3 subjects (Figure 5A). One of these subjects also shared a TCR junction between different visits, suggesting clonotype persistence.

Although there was no junction sharing between the few subjects in this study, we sought to determine if these shared TCRs were public (found in other individuals) or private specificities. Since our bulk profiles do not provide TCR pairing information, we were unable to directly determine antigen specificity of these TCRs. We therefore performed sequence comparisons by BLAST analysis against the National Center for Biotechnology (NCBI) nonredundant protein database (https://blast.ncbi.nlm.nih.gov/Blast.cgi) (Supplemental Table 2). We found that rearranged TRAV (TRAV26-2-CILPLAGGTSYGKLTF) and TRBV (TRBV7-8-CASSLGQAYEQYF) chains detected in donor 1 at week 104 perfectly matched a well-characterized immunodominant TCR recognizing EBV (39). Using PCR analysis, we found measurable EBV DNA in peripheral blood from this donor at week 104, but not week 52, suggesting that failure to control EBV was associated with likely expansion of EBV-specific CD8^+^ TEX cells. Together, our results suggest that the CD57^+^ and PD-1^+^ populations shared common precursors in some subjects.

We next directly compared PD-1^+^ and CD57^+^ T cells by DGEA to distinguish the populations from one another. PD-1^+^ T cells expressed high levels of the genes *CD28*, *IL2*, *CD27*, and *PDCD1*, while CD57^+^ T cells expressed higher levels of NK cell receptor (NKRs) genes, including *FCGR3A*, *LILRB1*, *KLRD1*, and multiple iKIR genes (*KIR2DL1*, *KIR2DL2*, *KIR3DL1*, *KIR3DL2*; Figure 5B). iKIRs are membrane proteins with inhibitory isoforms that bind HLA class I molecules (40). Signature genes from CD57^+^ T cells comprised a highly interconnected network of genes classically found in NK cells, but also in terminal effector CD8^+^ T cells (41) (Figure 5C).

GO analysis of the genes more highly expressed in CD57^+^ T cells showed enrichment of terms related to NK cell function, as well as cytotoxic functions characteristic to both NK and CD8^+^ T cells (Figure 5, D and E). This gene signature suggests functional similarities between the response-associated CD57^+^ CD8 cells and NK cells. In contrast, genes more highly expressed in PD-1^+^ T cells were enriched for annotation terms suggesting T cell costimulation (*CD28*, *CD40LG*), proliferation (*IL2*), and differentiation (*IL23A*, *FOXP3*; Figure 5, F and G). Notably, the elevation of these activation-related genes in the PD-1^+^ T cells is relative, and although they appeared to be more active than the CD57^+^ T cells, they had lower expression of activation pathways and reduced proliferative capacity compared with nonnaive and populations with exhausted-like phenotypes (Figure 4).

Finally, to further assess the differences in functional pathways between PD-1^+^ and CD57^+^ T cells, we compared their enrichment for 111 previously defined gene sets using gene set enrichment analysis (GSEA) (42). Several highly overlapping cytotoxicity-associated modules, including GZMB.mod and CD244.mod, were enriched in CD57^+^ T cells relative to PD-1^+^ T cells. Modules associated with T cell activation, inhibition, and differentiation, such as CD28.mod and CTLA4.mod, were more highly enriched in PD-1^+^ T cells (Supplemental Figure 15).

## Discussion

Using a combination of transcriptomics, cytometry, and functional assays, we performed a detailed analysis of the phenotype and function of residual and recurrent CD8^+^ T cells following alefacept therapy in RO T1D subjects. This systems-level approach identified a response-associated CD8^+^ T cell signature that was defined by activation- and exhaustion-associated gene expression and higher frequency of 2 related, but unique, CD8 memory phenotypes. These IR-expressing cells expressed features of exhaustion but were phenotypically distinct from one another, distinguishable by high reciprocal expression of PD-1 or CD57. The divergent inhibitory phenotypes of these cells, including iNKR expression by the CD57^+^ T cells, point to a possible role for multiple inhibitory systems driving effector CD8^+^ T cells toward an inhibited or exhausted state that could be beneficial in the context of T1D and other autoimmune diseases.

We show that the sorted CD57^+^ and PD-1^+^ T cells shared some rearranged TCR sequences, a commonly used measure of T cell clonality. This finding suggests that CD57^+^ and PD-1^+^ T cells shared a common lineage. However, because of the limited scope of our observations, we are unable to discriminate between different models for how CD57^+^ and PD-1^+^ T cells are derived from a common ancestral precursor. Larger studies utilizing single cell RNA-seq and samples taken over time will be required to clarify these lineage relationships. Such studies may also clarify TCR pairing and antigen specificity of exhausted cells. Integrating knowledge of lineage relationships and antigen specificity will lead to a fuller understanding of the relationship of exhausted cells in T1D progression and response to therapy.

The hypoproliferative DP cells described in the AbATE trial (22, 43) were defined by their expression of the transcription factor EOMES, effector molecules, and multiple IRs, including TIGIT and KLRG1, and they were found to expand after treatment. The subtypes of CD8^+^ T cells associated with response to alefacept, however, were distinct from these DP cells. First, the population dynamics differed in that clusters 12 and 20 did not expand from baseline frequencies but, rather, were maintained or recovered to baseline levels after treatment in responders as compared with nonresponders. Therefore, while both drugs may induce or spare a favorable, tolerogenic CD8^+^ T cell phenotype, the dynamics of these cells may reflect differences in drug mechanisms. Teplizumab targets CD3 and, as an agonist of the receptor, could activate cells and thereby induce exhaustion. In contrast, alefacept targets CD2^hi^ cells and therefore depletes activated effector memory subsets. It is possible that alefacept also acts by inhibiting costimulation by blocking CD2 signaling, thereby inducing functional inhibition rather than depletion (15). Tregs were also spared, so this — combined with functional inhibition of effector cells — may have promoted a more tolerogenic immune state in alefacept responders. We note, however, that we have not detected Treg signatures in either previous (22, 24) or the present studies of biologic therapies in T1D.

The CD57^+^ T cells expressed features of cytotoxicity not seen in canonically exhausted cells. Expression of CD57 on the surface of CD8^+^ T cells increases during T cell differentiation and is considered a marker of cytotoxic function (44–46). CD57 is expressed by heterogeneous populations of memory T cells and terminally differentiated effector T cells (45–47). CD57^+^CD8^+^ T cells have been described as senescent in chronic HIV infection (47) and in other diseases with chronic immune stimulation such as rheumatoid arthritis and in transplantation (44–46). During chronic viral infections, senescent T cells express CD57, KLRG1, and killer cell immune globulin-like receptors and are capable of producing a significant amount of effector cytokines (48). In T1D, elevated expression of CD57 and CD95 has been described in β islet cell–specific CD8^+^ T cells of patients with newly diagnosed T1D compared with healthy controls, and these cells are present in subjects with higher levels of C-peptide (49), raising the possibility that these cells participate in a protective, rather than pathogenic, role. In a follow-up study, the same group showed that change in β cell–specific CD8^+^ TEM cells expressing CD57 was positively correlated with C-peptide change in subjects younger than 12 years of age. Autoreactive CD57^+^ effector CD8^+^ memory T cells bore the signature of enhanced effector function (higher expression of Granzyme B, killer-specific protein of 37 kDa, and CD16 and reduced expression of CD28) compared with their CD57^–^ counterparts, and network association modeling indicated that the dynamics of β cell–reactive CD57^+^CD8^+^ TEM cell subsets were strongly linked (50). These results suggest that cytotoxic, CD57^+^CD8^+^ TEM cells may play a protective role during the early stages of T1D and in subjects who maintain higher C-peptide over time. However, they have not been identified in all subjects with high or maintained levels of C-peptide (26).

Alternatively, the protective effect of CD57^+^CD8^+^ T cells observed here and in previous trials could reflect the loss of cytotoxic function in a subset of terminal, cytotoxic CD57^+^ T cells. Increased expression of inhibitory receptors, including iNKRs, concurrent with a reduction in activation markers like CD28 and HELIOS, as well as loss of proliferative capacity, could be indicative of the expansion or maintenance of a dysfunctional, exhausted-like CD57^+^CD8^+^ T cell subset in subjects with better outcomes in T1D. Our observation of multiple phenotypically distinct CD57^+^ clusters by CyTOF analysis (Figure 3A) suggests that CD57 marks multiple functionally discrete cell types spanning a range of functional ability and that cytotoxic function itself may not be the protective mechanism at play in this and other trials.

The higher frequency of cluster 12 in nonresponders at baseline may suggest that a higher frequency of CD57^+^ exhausted-like CD8^+^ T cells is predictive of a negative outcome. Alternatively, the consistent frequency of cluster 12 in responders over time suggests that maintenance of a small terminal CD57^+^ population, as opposed to the depletion of it, may be beneficial to therapy response and outcome. A beneficial role for CD57^+^CD8^+^ T cells is also supported by the manual gating analysis of GZMB^+^ and CD57^+^ CD8^+^ T cells (Figure 2) that showed a slight increase in the frequencies of these populations over time in responders versus nonresponders.

Although it is not evident why nonresponders underwent depletion of cluster 12 cells and responders did not, it is possible that these cells expressed higher levels of CD2 in nonresponders than in responders, making the cells more prone to depletion by alefacept. The maintenance of gated CD57^+^ and GZMB^+^ TEM cells in placebo-treated subjects (Figure 2) suggests that the reduction seen in these populations is specific to alefacept treatment as opposed to natural disease progression. Further experimentation is needed to clarify the phenotypic or functional differences that might have contributed to differential depletion of cluster 12 cells between response groups in this trial.

Recent studies have identified subtypes of TEX cells defined by their differentiation state and their proliferative and functional abilities. Precursor exhausted cells retain their proliferative potential, whereas terminally exhausted cells have lost their ability to divide, as well as all functional capability. In addition to loss of function and proliferative capacity, the trajectory from a precursor to terminally exhausted state is characterized by changes in surface marker and transcription factor expression, including increased expression of PD-1 and decreased expression of activation receptors. The PD-1^+^ T cells defined here resemble the stem-like (51), precursor (52) or progenitor (53) exhausted cells identified by others, in that they are hypoproliferative and PD-1^hi^, but appear to retain some activation-associated functions. The CD57^+^ T cells defined here, however, do not clearly fall along previously described trajectories, as neither CD57 nor iKIRs are present in T or NK cells in mice (54), where much of the fundamental work on exhaustion has been done. The expression of cytotoxic markers by CD57^+^iKIR^+^ T cells may indicate that they represent a distinct lineage of exhausted-like cells.

PD-1/PD-L1 ligation inhibits TCR signaling by preferentially dephosphorylating CD28 (55, 56). The PD-1^+^ T cells in this study maintained a high level of CD28 gene expression and, thus, would be expected to remain sensitive to inhibition by PD-1/PD-L1 signaling. In contrast, upregulation of iKIRs on the CD57^+^CD28^–^ cells could reflect an alternative, CD28-independent inhibitory mechanism. In other studies, upregulation of NKRs, including iKIRs, was observed in CD8^+^ T cells following loss of CD28, and these receptors were shown to provide a costimulatory signal to prolong CD8^+^ T cell survival and function (41). The CD57^+^ T cells described here could represent a terminally exhausted phenotype deriving directly from the PD-1^+^ T cells after they have lost CD28 expression. The functional consequences of these CD57^+^, postexhaustion cells on disease and outcome could be determined by the quality (i.e., inhibitory or activating) of NKR upregulation that occurs following loss of CD28. Inhibitory KIR expression, as observed here, would contribute to an inhibitory and, thus, potentially protective immune state for autoimmune disease.

The reciprocal expression pattern of *PDCD1* and iKIRs in CD8^+^ T cells has been noted previously. Duraiswamy et al. (57) described the striking observation that KIRs and killer cell lectin–like receptor (KLRs) were completely downregulated on PD-1^hi^ cells. Boelen et al. (58) discussed similarities between the iKIR-HLA receptor-ligand system and the PD-1–programmed death ligand 1/2 system. Both iKIR and PD-1 are inhibitory receptors that block proximal TCR signaling and are upregulated in the context of chronic viral infection and on tumor-infiltrating lymphocytes. Our results support these previous studies and extend them into the realm of therapies for T1D — and perhaps other autoimmune diseases.

iKIRs can affect T cell responses indirectly through NK cells or directly via expression on CD8^+^ T cells (58). Recent evidence shows that functional engagement of iKIRs by their MHC ligands enhances clinical CD8^+^ T cell responses against HIV-1, HCV, and HTLV-1 viral infections (58). Other studies have shown a role for KIRs and IRs in EBV infections, such that in later stages of persistent infection, protective immunity to EBV may be reduced due to the preferential accumulation of hyporesponsive EBV-specific CD8^+^ T cells (59). These findings are consistent with our demonstration of expanded EBV-specific TCRs in CD57^+^CD8^+^ T cells, although in autoimmunity, hyporesponsiveness is clinically beneficial. It is important to note that our observations were made at the transcriptome level and that a fuller understanding of the functional implications of iKIRS in autoimmune disease progression will require additional experimentation with the protein products of these genes.

The statistical comparisons in this study were limited by small sample sizes, particularly in the analysis of discretized response (responder/nonresponder) where only 6–9 subjects were available per group at posttreatment time points. Future studies would benefit from a larger sample size to confirm and further characterize the role of exhausted or exhausted-like CD8^+^ T cells in T1D immunotherapy response.

Taken together, these studies support an association between IR-expression, hypoproliferative CD8^+^ T cells, and favorable outcome in T1D trials of immunomodulatory agents, although the mechanisms and molecular targets of initial immune perturbation differ with each agent used. This study also provides evidence that inhibitory NKR expression on hypoproliferative CD8^+^ T cells may be protective against the autoimmune response in T1D. Alefacept and other T1D therapies may therefore drive self-reactive T cells to a more exhausted state by promoting one or more differentiation pathway, thereby preventing further β cell destruction.

## Methods

### Study design and patients.

T1DAL was a phase-2, randomized, placebo controlled, double-blind clinical trial conducted at 14 clinical centers in the United States with a 9-month treatment period and 15 months of follow-up. Eligible participants were 12–35 years of age at the time of screening; < 100 days from diagnosis at the time of enrollment; positive for at least 1 diabetes-associated autoantibody (insulin, GAD-65, IA-2, ZnT8, or ICA); and had peak-stimulated C-peptide of > 0.2 nmol/L during a mixed meal tolerance test (MMTT) (11, 15). All subjects gave informed consent prior to enrollment in the trial. Eligible subjects were randomly assigned 2:1 to alefacept (33 patients) or placebo (16 patients). Participants received 15 mg alefacept (Amevive, Astellas) or equivalent volume of saline (placebo) i.m. weekly for 12 weeks and, after a 12-week pause, 12 additional weekly doses of alefacept or placebo. Participants underwent a 4-hour MMTT at screening, at 52 weeks, and at 104 weeks; a 2-hour MMTT at 24 and 78 weeks; and intensive diabetes management throughout. Additional participant and outcome data from the T1DAL trial are available online at (https://www.itntrialshare.org/T1DAL.url). PBMCs were collected from 30 alefacept-treated and 12 placebo-treated patients at weeks 0, 11, 24, 35, 52, 78, and 104 for flow cytometry and at weeks 0, 24, 35, 52, and 104 for RNA-seq and CyTOF (Figure 1A), followed by functional studies on selected samples. Responders were defined as participants that maintained or increased their baseline 4-hour C-peptide AUC (*n =* 9). Nonresponders were defined as participants that had greater than 40% loss of their 4-hour baseline C-peptide AUC values at 2 years (*n =* 9), consistent with prior ITN studies (15, 60).

### Flow cytometry immunophenotyping.

Flow cytometry analysis was run on 39 subjects (responders = 7, nonresponders = 9, partial responders = 11, and placebo = 12) from 7 visits (weeks 0, 11, 24, 35, 52, 78, and 104). Cryopreserved PBMCs from all subjects were thawed, incubated with FcX Block and stained with X-trial T and NK cell flow cytometry panels used in previously reported ITN studies (22, 43, 61) (Supplemental Table 3). Instrument standardization was performed using 8 peak rainbow calibration beads (Spherotech) adjusting photomultiplier tube (PMT) voltages for consistent seventh-peak mean fluorescence intensities. All samples from the same subject were run on the same day, and an internal control arm from the same subject was run each week. An average of 580,000 live lymphocyte events were collected per sample on a BD Fortessa using Diva software, and data were analyzed using FlowJo Mac Version 9.4 (Tree Star Inc). Gated populations with < 100 events were excluded from analysis.

### T cell proliferation assay.

Cryopreserved PBMCs from 4 alefacept-treated subjects at weeks 52 or 104 were thawed and sorted into nonnaive (CD45RA^–^), PD-1^+^, and KLRG1^+^TIGIT^+^ nonnaive CD8^+^ T cells and labeled with cell trace violet (Thermo Fisher Scientific). The labeled cells were mixed back with unlabeled autologous PBMC and stimulated in vitro with plate-bound anti-CD3 and anti-CD28 mAbs for 3 days (Supplemental Table 5). Proliferation was measured by Ki67 expression within all CD8 populations. Representative plots for CD8 populations and the gating strategy for Ki67 are shown in Supplemental Figure 12. The staining panels for sorting and proliferation are shown in Supplemental Tables 4 and 5. Flow acquisition and analysis was performed as described above.

### RNA-seq.

Out of 33 alefacept-treated subjects, 3 were lost to follow-up by week 104. Samples from the remaining 30 treated subjects (weeks 0, 24, 35, 52, and 104) were analyzed by RNA-seq. Bulk CD8^+^ T cells were FAC sorted and analyzed by RNA-seq as previously described (22). Low-quality libraries (median CV of coverage > 0.9, total reads < 5 million) were excluded, leaving 26 subjects with libraries available from downstream analysis (Supplemental Table 1). Counts were then normalized and log_2_ transformed, followed by batch correction using the limma R package (62). Five placebo subjects were included in the week 104 RNA-seq analysis, and the 3 placebo samples that remained after quality control (QC) filtering were included in the WGCNA analysis to maximize the sample size for module detection. The top 5000 most variable genes (ranked by coefficient of variation) were used to generate WGCNA modules using the *blockwiseModules* function from the WGCNA R package (34, 63). WGCNA was run with a soft thresholding power of 5, a signed network, a minimum module size of 50, and a clustering dendrogram cut height of 0.30. Module eigengenes were calculated using the *moduleEigengenes* function in the WGCNA R package (34, 63), and Pearson’s correlation was calculated between these eigengenes and other parameters, including clinical outcomes, CyTOF marker levels, and CyTOF cluster frequencies. GO analysis was performed using the *goana* function in the limma R package (62). String-db (https://string-db.org/) was used to find connected gene networks, and Cytoscape was used for gene network visualization (64).

Sequences of rearranged TCR chains, which include nontemplated nucleotides in the CDR3 junction, not present in the reference genome, were identified from genome-independent (de novo) assemblies of overlapping DNA segments (38, 65). Pilot spike-in experiments showed that rearranged TCRs could be reassembled from high-abundance clonotypes in bulk RNA-seq T cell profiles, but not from monocyte profiles.

### Mass cytometry (CyTOF) staining and analysis.

Thawed cryopreserved PBMC were stained for viability using cisplatin (Enzo Life Sciences) prior to staining with a surface antibody cocktail (Supplemental Table 6). Samples were then washed, fixed using the Maxpar Nuclear Antigen Staining Buffer (Fluidigm), and stained with an intracellular mAb cocktail. Samples were stored with 125 nM MaxPar Intercalator-Ir (Fluidigm) in Fix and Perm Buffer (Fluidigm) at 4°C overnight or up to 1 week prior to acquisition. For acquisition, cells were resuspended (0.5 × 10^6^/mL) in cold ultrapure water containing one-fifth EQ Four Element Calibration Beads (Fluidigm) and were acquired at a rate of 300–500 events/second on a CyTOF1.5, with upgrades (Fluidigm) running CyTOF software version 6.0.626 and using a Super Sampler system (Victorian Airship & Scientific Apparatus). Files were converted to .FCS and then randomized and normalized for EQ bead intensity using the CyTOF software. FlowJo software (version 10.4) was used to manually gate and export FCS files of CD8^+^ T cells.

Gated CD8^+^ T cells were analyzed in R using the Cytofkit and flowCore packages (66). Events were down-sampled to include the same number of events from each subject at each time point, and they were then combined for all downstream analyses. Intensity values were arcsinh-transformed with a cofactor of 5 prior to analysis. Markers that were used for preliminary gating and those with a high CV were excluded from dimensionality reduction and clustering. The full set of markers used to generate t-SNE plots and clusters was: CCR7, CD38, CXCR3, CD27, CD45RA, CD45RO, CD57, CD25, TIGIT, EOMES, T-BET, CD95, HELIOS, KLRG1, TIM3, PD-1, CD122, 24B, CD161, CD127, and NKG2D. Rphenograph (67) was used to generate clusters from downsampled files.

### Statistics.

Longitudinal flow cytometry and RNA-seq data were analyzed by repeated-measures 1-way ANOVA. Group comparisons were made by comparing their least square means at each visit. Mann-Whitney *U* test was used for comparison between 2 distinct groups, and within-group comparisons were performed by Wilcoxon signed-rank test. C-peptide slopes were calculated by fitting a random effects linear model over all visits per subject as previously described (68).

Group comparisons of C-peptide data at primary endpoint were analyzed by fitting an ANCOVA model with change from baseline as the outcome and baseline value as a covariate. FDR or Bonferroni correction were applied where appropriate to adjust for multiple comparisons for statistical tests. All comparisons required the level of significance to be kept at α = 0.05 for 2-sided tests. SAS version 9.4 was used for all data analyses, and graphs were produced in R (https://www.R-project.org). Data sets for these analyses are accessible through TrialShare, a public website managed by the ITN (https://www.itntrialshare.org/T1DAL.url), and the GEO Repository (accession GSE158292). Code and data files used in figures are deposited at GitHub (https://github.com/BenaroyaResearch/Diggins_Serti_Linsley_JCI_Insight_2020; commit ID 918a8972f9a87842a4385966491320a9f5afd184).

To determine correlations between gene expression and the frequency (percentage) of specific cell types identified by CyTOF, Pearson’s correlation values were calculated between blue module eigengene values and cell-type frequencies from Rphenograph. Student’s asymptotic 2-tailed *t* tests were then used to calculate the significance of the correlations. A *P* value less than 0.05 was considered significant. Data are presented as mean ± SEM.

### Study approval.

Protocols for these studies were approved by the IRB of Benaroya Research Institute. Protocols for the T1DAL clinical trial were approved under the auspices of NCT00965458 (11). All participants or parents provided written informed consent or assent (<18 years old) (15).

## Author contributions

KED and ES performed experiment design, data analysis, figure generation, and manuscript writing. VM assisted with data analysis and manuscript edits. MR assisted with data analysis. TL performed statistical analysis, figure generation, and data management and acted as consulting statistician. EB assisted with data analysis. GN provided guidance on experiment design, figure design, and data management. SAL and PSL contributed to experiment design and execution, data analysis, data management, and manuscript writing.

## Supplementary Material

Supplemental data

## Figures and Tables

**Table 1:**
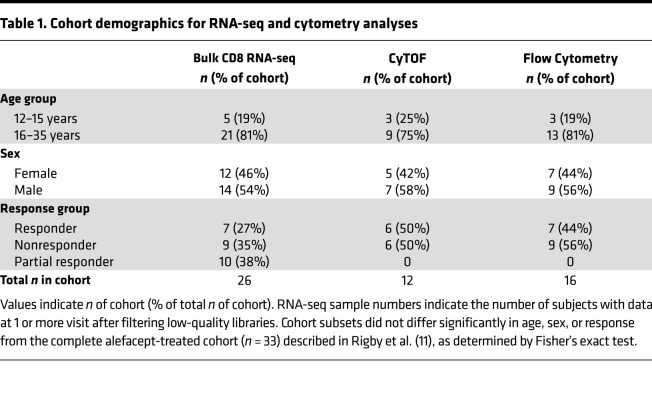
Cohort demographics for RNA-seq and cytometry analyses

## Version 1 12/22/2020

In-Press Preview

## Version 2 02/08/2021

Electronic publication
