# Supplementary material for: Exhausted-like CD8+ T cell phenotypes linked to C-peptide preservation in alefacept-treated T1D subjects
Source: JCI Insight. 2021 Feb 8;6(3):e142680. doi: 10.1172/jci.insight.142680 (PMC7934874; doi:10.1172/jci.insight.142680)
Supplement: Supplemental data [file jciinsight-6-142680-s147.pdf]

Supplementary Materials: Exhausted-like CD8 T cell phenotypes linked to C-peptide preservation in alefacept-treated T1D subjects

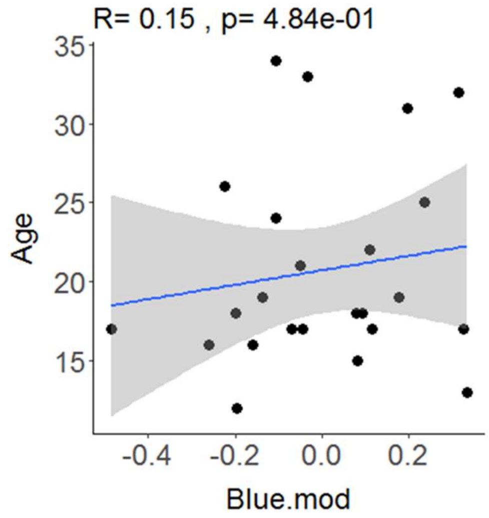

Figure S1. Blue module gene expression was not correlated with age.

Pearson correlation between subject age and blue module eigengene expression at week 104. P-value from linear model: eigengene ~ age; statistic summarized by 1- way ANOVA.

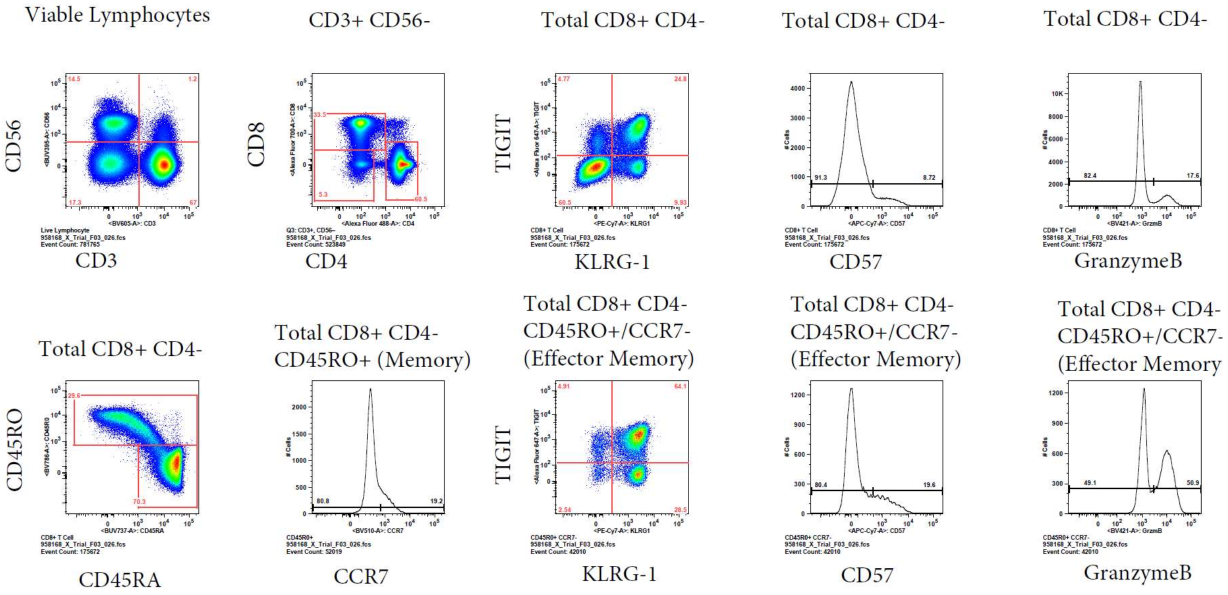

Figure S2. Representative gating of CD8 T cells (top) and memory CD8 T cell subpopulations shown in Figure 2 (bottom). CD8 gate excluded CD4+ and CD56+ cells. Representative gates and population histograms from a single subject are shown for DP (KLRG1+TIGIT+), CD57+, and Granzyme B+ TEM populations.

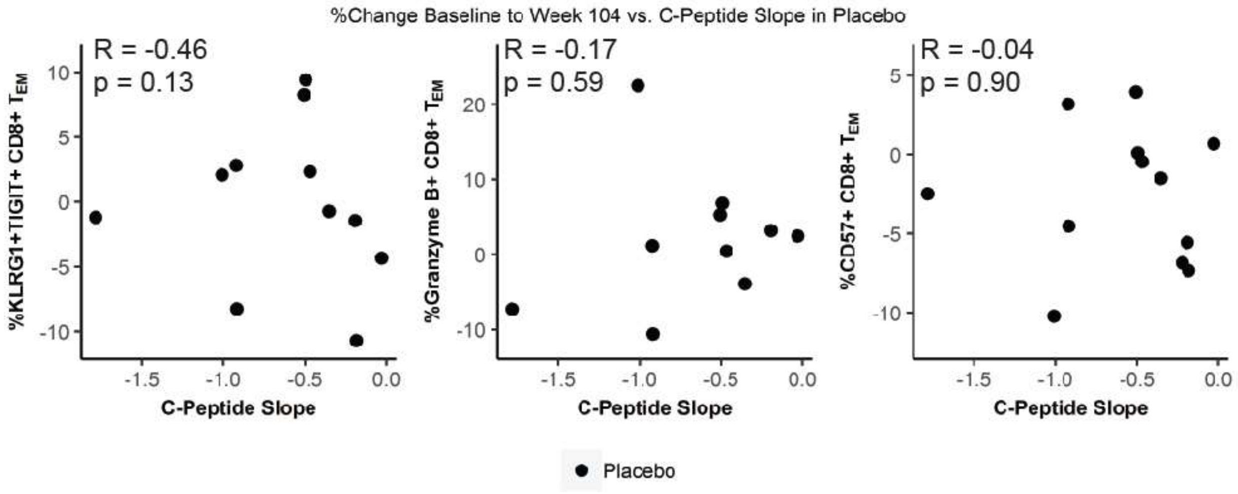

Figure S3. Placebo correlations between TEM subsets and c-peptide change (slope) at week 104. Spearman correlation and correlation p-value are shown.

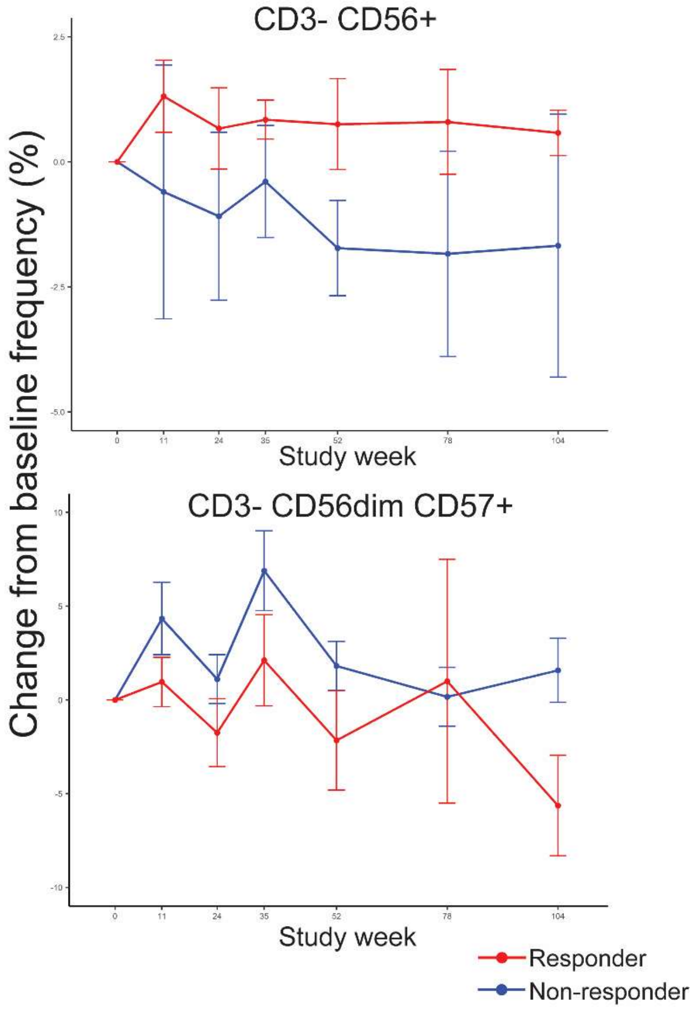

Figure S4. NK cell frequencies did not vary by response over the course of the trial. Change in subset frequency of parent population, shown for NK cells (CD3- CD56+; % of lymphocytes) and CD57+ NK cells (CD3- CD56dim CD57+; % of CD56+) from flow cytometry analysis, gated and analyzed as in Figure 2. Repeated measures 1-way ANOVA was used for significance calculations. No significant differences existed between responders and non-responders. P-values < 0.05 were considered significant.

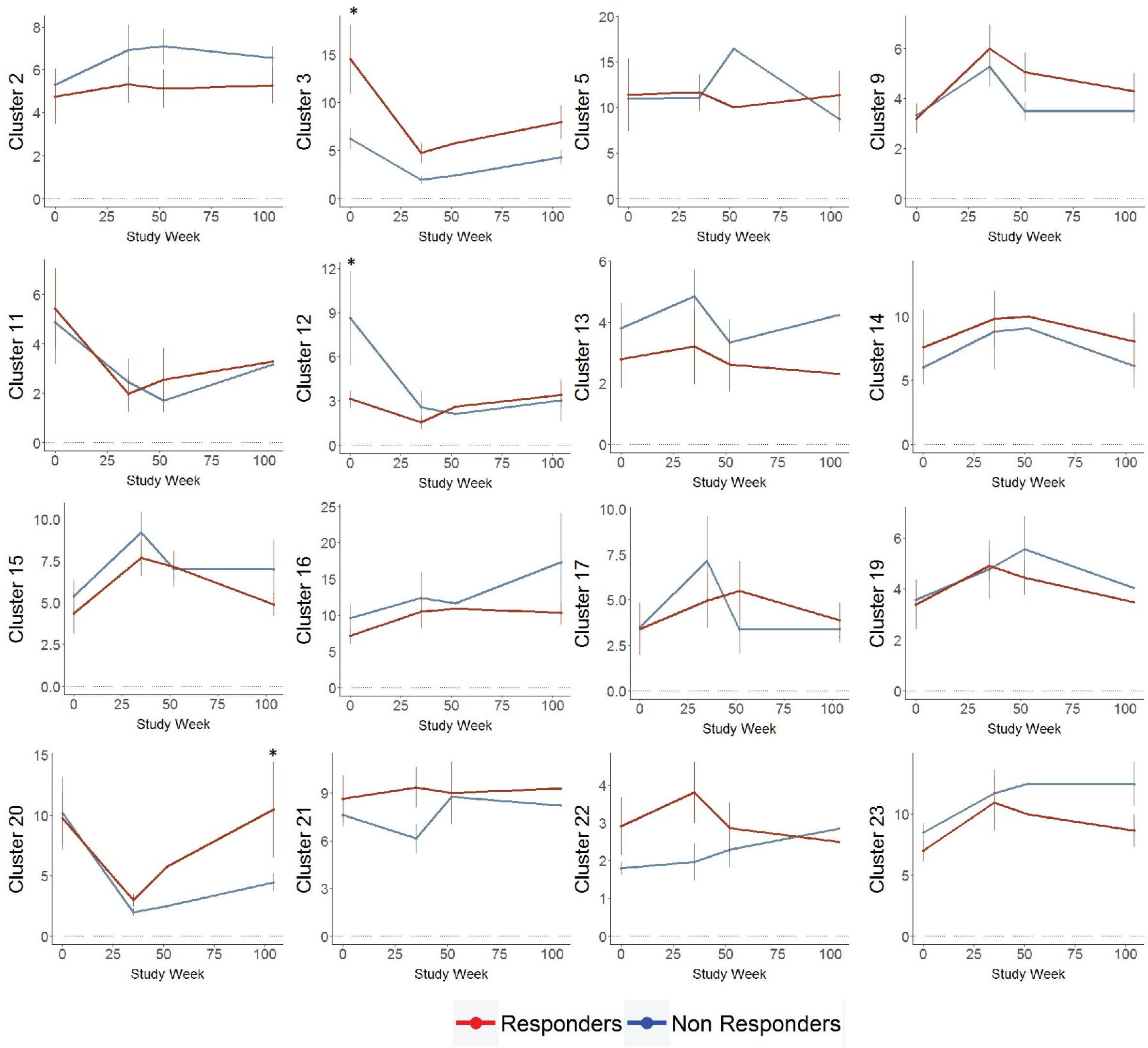

Figure S5. CyTOF clusters change in frequency over time by response group. Cluster frequency for all clusters containing >1% of total cells. Significance of response differences tested by repeated-measures 1-way ANOVA; n=12; p-values < 0.05 were considered significant.

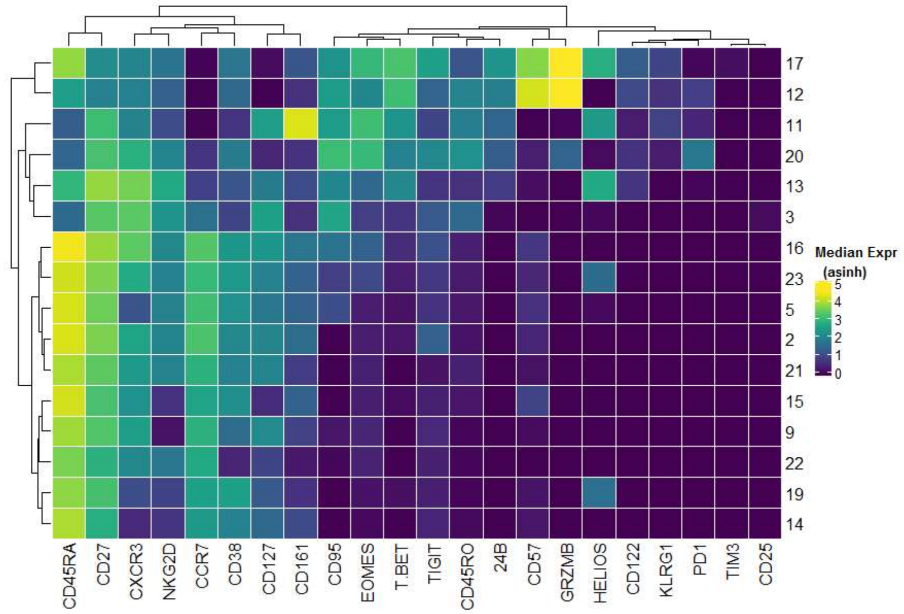

Figure S6. Median marker expression of CyTOF clusters. Median intensity values were transformed to an arcsinh scale with cofactor 15, and median expression was calculated across subjects.

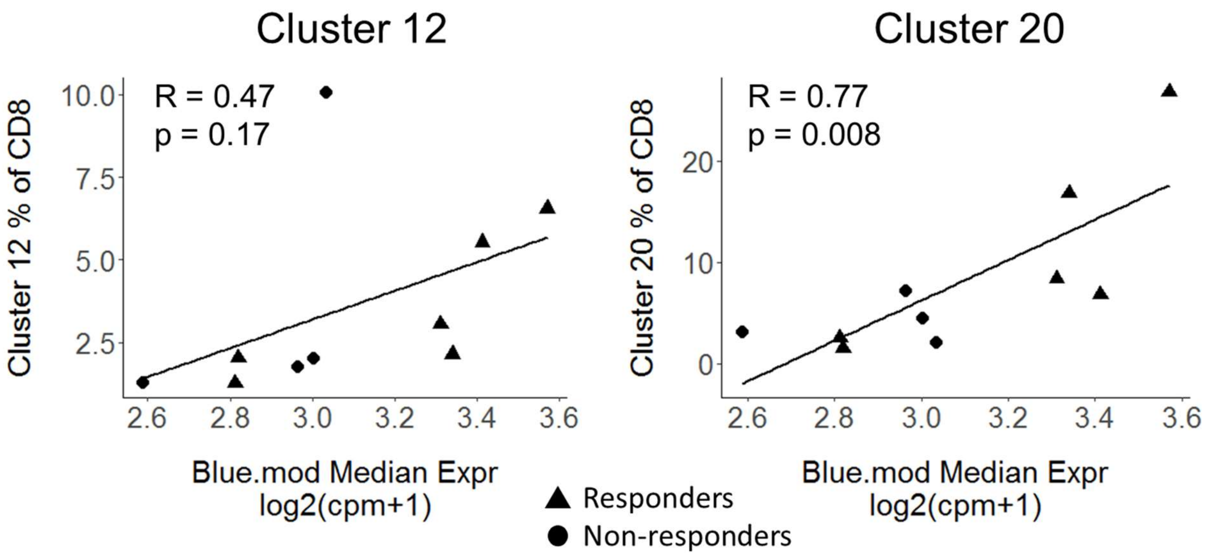

Figure S7. Cluster 12 and 20 frequencies were correlated with blue module gene expression at week 104. Percentage of cells in Cluster 12 and Cluster 20 plotted against median blue module gene expression at week 104. Pearson correlation and the corresponding 2-tailed t test were performed using cor.test function in R.

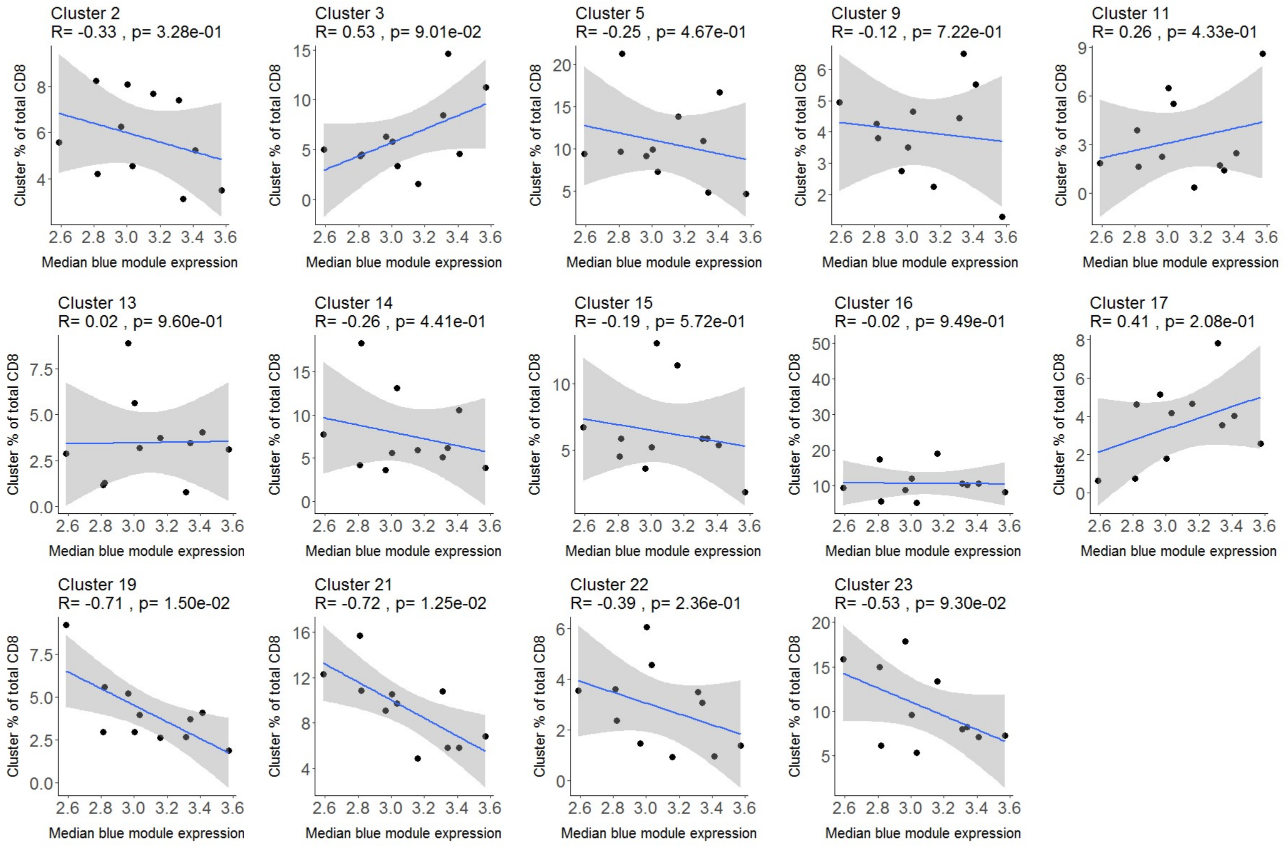

Figure S8. Cluster frequency correlations with median blue module expression at week 104.

Median blue module gene expression was calculated and correlated with each cluster's % of total CD8 for week 104 samples. Pearson correlation and the corresponding 2-tailed t test were performed using cor.test function in R.

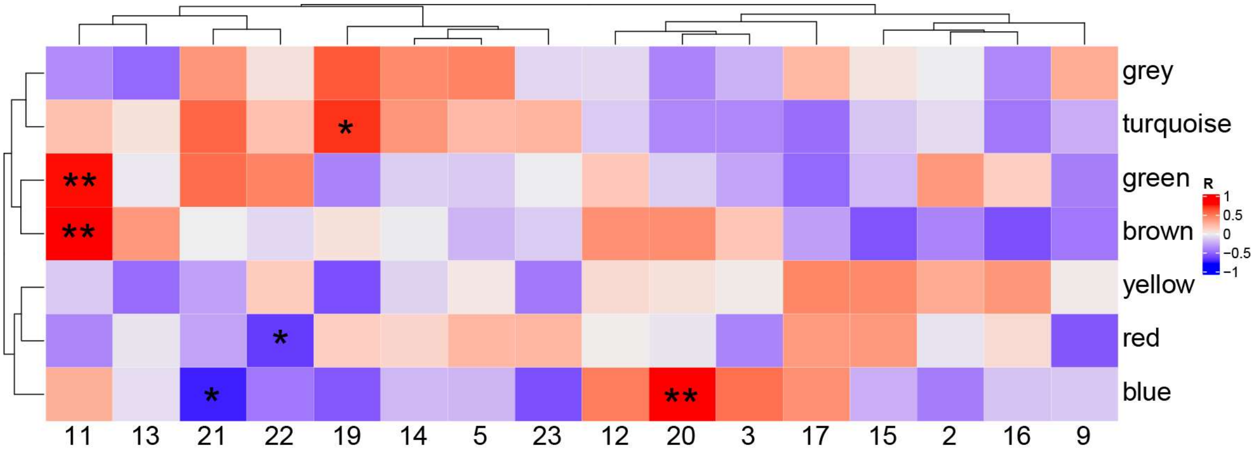

Figure S9. Cluster correlations with median module gene expression. Pearson correlation was calculated between week 104 cluster % of total CD8 and median expression of module genes (log2(cpm+1)). Correlations that were significantly different from zero as determined by a two-sided t-test are labeled with asterisks (* < 0.05; ** < 0.01).

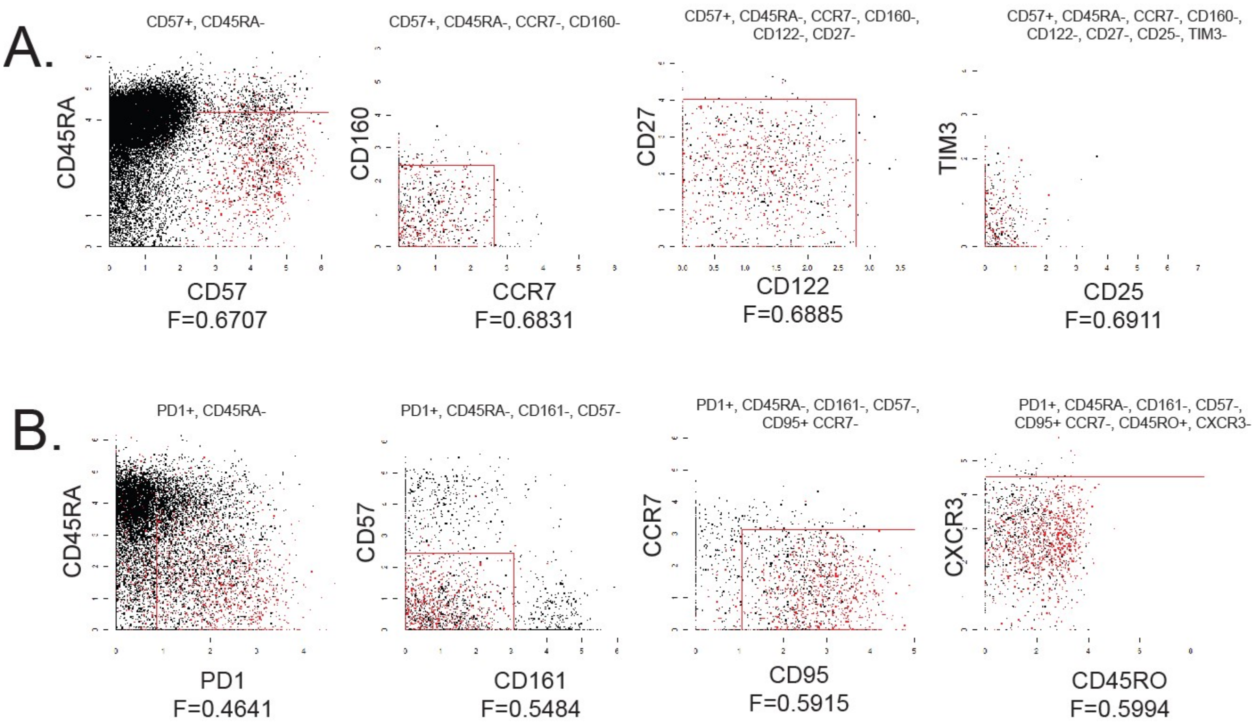

Figure S10. Hypergate analysis for sorting marker selection. A) Hypergate's suggested gating scheme for cluster 12 and B) for cluster 20. F score indicates predicted population purity for each cumulative set of gates.

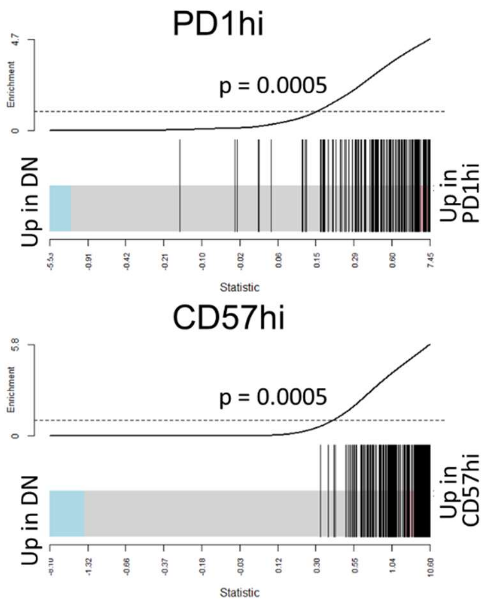

Figure S11. Clusters 12 and 20 were enriched for DP genes relative to DN population. DP genes were defined by contrasting sorted TIGIT+KLRG1+ (DP) and double negative cells by differential gene expression analysis and selecting genes that were significantly upregulated in DP relative to DN cells (adjusted $p < 0.05$). Full gene list are provided in supplementary file. Analysis was performed in R with the barcodeplot function for visualization and the non-competitive rotation gene set test *roast* for enrichment p-values.

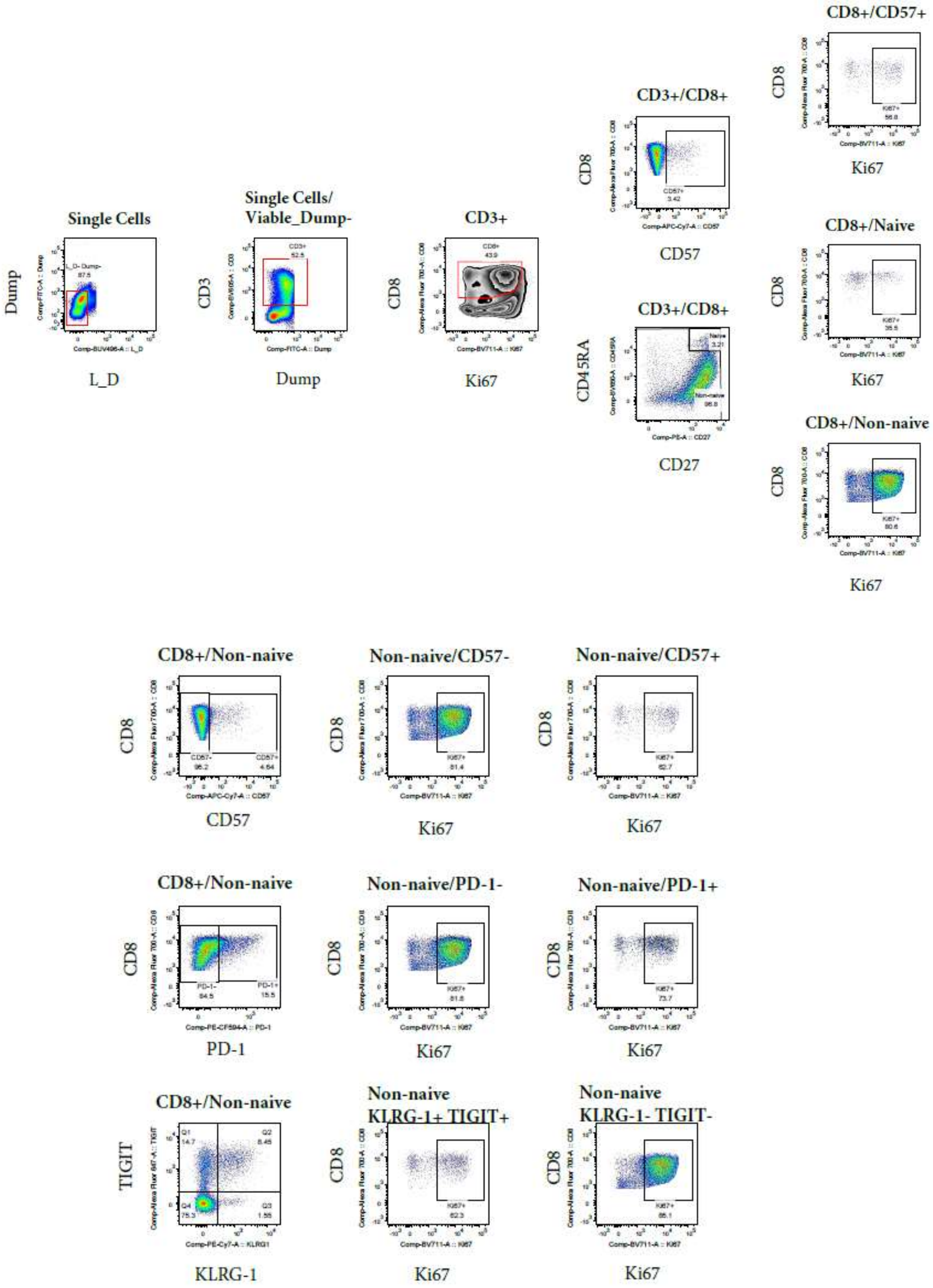

Figure S12. Representative gating of PD-1+, CD57+ and KLRG1+TIGIT+ non naïve (CD45RA-) CD8 T cells subsets (top) and Ki67 expression shown in Figure 4D (Bottom graphs).

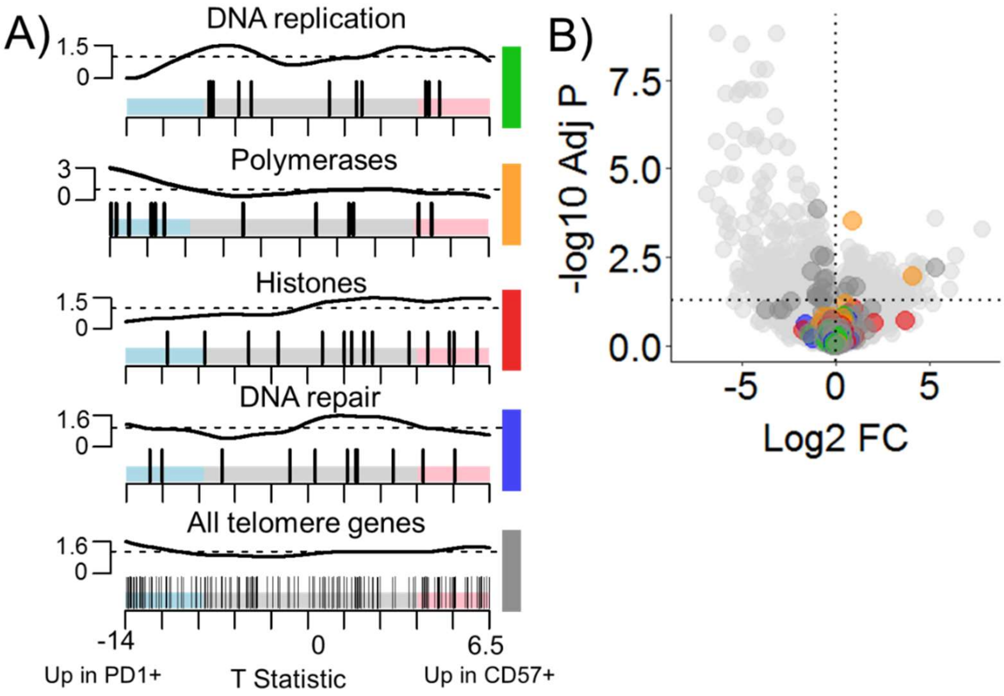

Figure S13. CD57+ cells were not enriched for telomere and DNA maintenance genes relative to PD-1+ cells. CD57+ cells and PD1+ cells were contrasted using limma for DGEA as in Figure 5. **A)** A set of genes related to replicative senescence was defined as the intersection between the GO_TELOMERE_ORGANIZATION gene set from the Molecular Signatures Database (MSigDB) and REACTOME database pathways. The overlapping gene set (n=175) included DNA replication (RPA1, RIF1, etc), polymerases (POLD1, PARP1, etc), histones (HIST1H3A, HIST1H4A, etc.), and DNA repair (ATM, XRCC1, etc). Gene subsets were analyzed by non-competitive gene enrichment analysis *roast* in R and visualized with the barcodeplot function in the limma package. Only the subset of polymerase genes showed significant enrichment ($P < 0.05$), with increased enrichment in PD1+ cells relative to CD57+ cells ($p = 0.01$). **B)** Volcano plot shows differential expression of telomere-related genes color coded by the gene sets defined in (A).

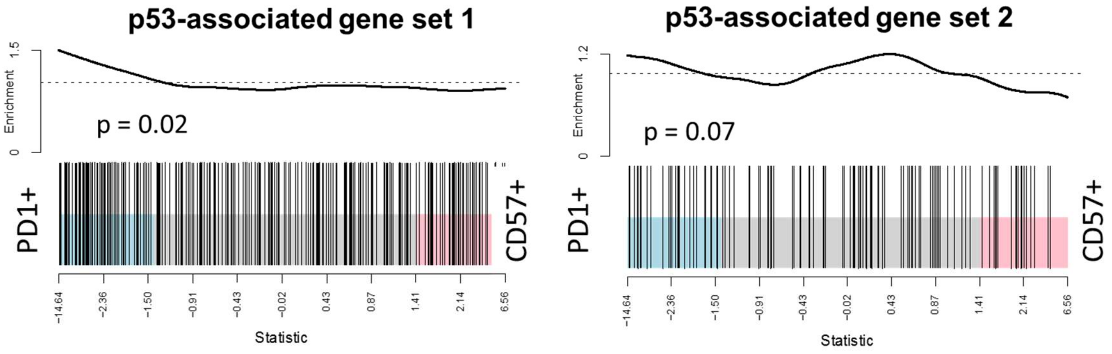

Figure S14. CD57+ cells were not enriched for p53-associated genes. CD57+ population was contrasted with PD1+ population using roast and barcodeplot in R to determine if CD57+ cells were enriched for senescence related genes. The gene sets tested included genes found to be targeted by or associated with p53 in previous studies. Gene set 1 (left): 346 p53 target genes identified across 319 studies by Fischer, *Oncogene* 2017. Gene set 2 (right): 116 top genes identified in at least 6 of 16 studies by Fischer, *Oncogene* 2017.

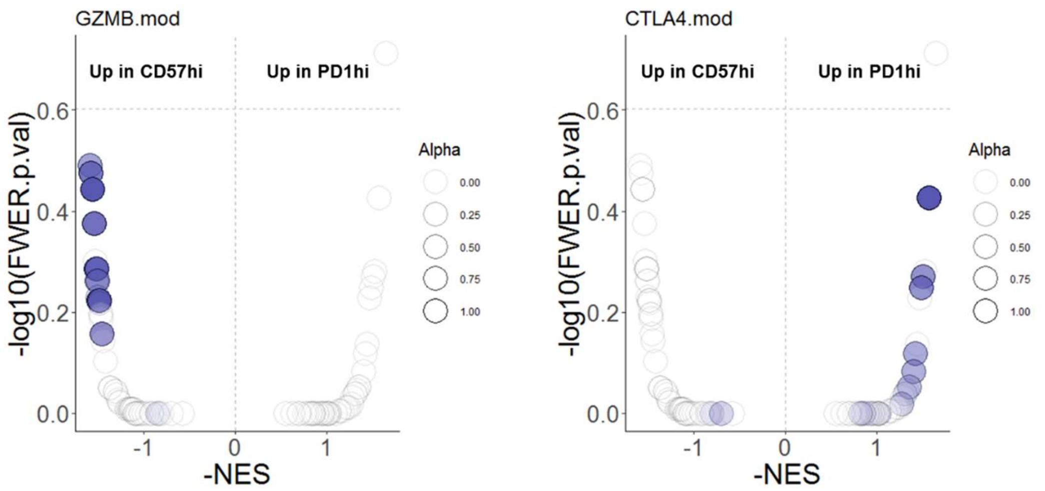

Figure S15. Gene sets related to cytotoxicity were enriched in CD57hi cells, while inhibitory-related modules were enriched in PD1hi cells. GSEA was run contrasting sorted CD57hi and PD1hi populations, using the 111 modules defined by Linsley, et al.. Nominal enrichment score of each module is plotted against its adjusted p-value, with CD57hi cells on the left and PD1hi on the right. The alpha color of each point indicates its overlap with the specific module (GZMB.mod and CTLA4.mod, respectively). Modules with high gene overlap are assumed to be functionally related, and similar enrichment of overlapping modules provides internal validation of functional enrichment.

Supplementary Table 1. Post-QC RNA-seq Sample Numbers

Week	Responder	Partial Responder	Non-responder
**0**	6	10	8
**24**	6	9	8
**35**	6	10	8
**52**	6	7	8
**104**	7	7	8

Table S1. Number of RNA-seq samples per response group and visit remaining after excluding low quality libraries. See Materials and Methods for exclusion criteria.

Supplementary Table 2. TCR junctions identified in sorted populations.

Lib.ID	Donor	Week	Cell.Type	Junction
lib36283	10213	52	CD57+	CASSSEATNEKLFF
lib36283	10213	52	CD57+	CVVSDGGNTPLVF
lib36283	10213	52	CD57+	CASSLGFGSYEQYF
lib36283	10213	52	CD57+	CILRDDNAGNMLTF
lib36285	10213	52	PD1+	CVVSDGGNTPLVF
lib36285	10213	52	PD1+	CASSLGFGSYEQYF
lib36285	10213	52	PD1+	CASSSEATNEKLFF
lib36285	10213	52	PD1+	CILRDDNAGNMLTF
lib36289	10458	52	CD57+	CSARNKEEGLANYGYTF
lib36295	10458	104	CD57+	CILPLAGGTSYGKLTF
lib36295	10458	104	CD57+	CSARNKEEGLANYGYTF
lib36295	10458	104	CD57+	CASSLGQAYEQYF
lib36295	10458	104	CD57+	CARTGRNTGELFF
lib36297	10458	104	PD1+	CASSLGQAYEQYF
lib36297	10458	104	PD1+	CILPLAGGTSYGKLTF
lib36297	10458	104	PD1+	CARTGRNTGELFF

Table S2. TCR junctions identified in sorted populations. Sequence comparisons were performed by BLAST analysis against the NCBI (National Center for Biotechnology) non-redundant protein database.

Target	Format	Clone	Vendor
CD56	BUV395	NCAM16.2	BD
CCR7	BV510	G043H7	BL
CD4	BB515	RPA-T4	BD
CD3	eV605	OKT3	eBio
PD-1*	eV655	J105	eBio
TIGIT	APC	MBSA43	eBio
CD127 #	BV711	A019D5	BL
CD8	Alexa700	RPA-T8	BL
CD45RA	BUV737	Hl100	BD
KLRG-1	PE-Vio770	REA261	Miltenyi
CD57	APC-Vio770	TB03	Miltenyi
CD45RO	BV786	UCHL1	BD
GranzymeB	BV421	GB11	BD
Eomes	PE	WD1928	eBio
FOXP3	PE-CF594	259D	BD

Table S3. Flow cytometry panel for immunophenotyping and intracellular staining.

Supplementary Table 4. Cell sorting panel			
Target	Format	Clone	Vendor
CD56	APC-Cy7	HCD56	BL
CD19	APC-Cy7	HIB19	BL
CD14	APC-Cy7	M5E2	BL
CD8	Alexa700	SK1	BL
CD3	BV605	UCHT1	BL
PD-1	PE-cf594	EH12.1	BD
TIGIT	APC	MBSA43	eBio
KLRG-1	PE-Cy7	REA261	Miltenyi
CD45RA	BUV737	HI100	BD
CD57	BV421	NK-1	BD
CD27	PE	M-T271	BL

Table S4. Flow cytometry panel used for cell sorting of various CD8 memory subsets (shown in figure 4).

Supplementary Table 5. Proliferation / ICS flow panel			
**Target**	**Format**	**Clone**	**Vendor**
CD56	FITC	NCAM16.2	BD
CD45RA	BV421	HI100	BD
CD8	Alexa700	SK1	BL
CD3	BV605	UCHT1	BL
PD-1	BUV737	EH12.1	BD
TIGIT	APC	MBSA43	eBio
KLRG-1	PE-Vio770	REA261	Miltenyi
CD57	APC-Vio770	REA769	Miltenyi
CD107a	PE-Dazzle594	H4A3	BL
Ki67	BV711	B56	BD
Eomes	PE	WD1928	eBio
IL-2	BB700	MQ1-17H12	BD
IFN-γ	BUV395	B27	BD
TNFα	BV650	MAb11	BL

Table S5. Flow cytometry panel used in the proliferation assay (Ki67) and intracellular cytokine staining.

Mass	Target	Clone	Supplier	Cocktail
89	CD45	HI30	Fluidigm	Surface
141	CD45RO	UCHL1	Biolegend*	Surface
142	CD57	HCD57	Fluidigm	Surface
143	CD45RA	HI100	Fluidigm	Surface
144	CD38	HIT2	Fluidigm	Surface
145	CD8	RPA-T8	Biolegend*	Surface
146	CD4	RPA-T4	Biolegend*	Surface
147	KLRG1	14C2A07	Biolegend*	Surface
148	CD14	RMO52	Fluidigm	Surface
149	CD127	A019D5	Fluidigm	Surface
151	Helios	22F6	Biolegend*	Intracellular
152	CD160	BY55	Biolegend*	Surface
153	Tim3	F382E2	Fluidigm	Surface
154	CD3	UCHT1	Fluidigm	Surface
155	TIGIT	MBSA43	eBioscience*	Surface
156	CD25	M-A251	Biolegend*	Surface
158	CD27	L128	Fluidigm	Surface
159	CD161	HP3G10	Fluidigm	Surface
160	Tbet	4B10	Fluidigm	Intracellular
162	Eomes	WD1928	eBioscience*	Intracellular
163	CXCR3	G025H7	Fluidigm	Surface
164	CD95	DX2	Fluidigm	Surface
165	CD19	HIB19	Fluidigm	Surface
166	NKG2D	ON72	Fluidigm	Surface
167	CCR7	G043H7	Fluidigm	Surface
170	CD122	Tu27	Fluidigm	Surface
171	Granzyme B	GB11	Fluidigm	Intracellular
173	2B4	C1.7	Biolegend*	Surface
175	PD1	EH12.2H7	Fluidigm	Surface
176	CD56	NCAM16.2	Fluidigm	Surface
191/193	Iridium (cell size)	--	Fluidigm	Iridium
194-198	Cisplatin (viability)	--	Enzo Life Sciences	Cisplatin

*Unlabeled purified antibodies were conjugated to metal isotopes using Maxpar X8 Antibody Labeling Kits (Fluidigm) as per manufacturer's instructions.

Table S6. CyTOF panel for immunophenotyping and intracellular staining.
